# South-East Asia is the center of origin, diversity and dispersion of the rice blast fungus, *Magnaporthe oryzae*

**DOI:** 10.1111/nph.12627

**Published:** 2013-12-10

**Authors:** Dounia Saleh, Joëlle Milazzo, Henri Adreit, Elisabeth Fournier, Didier Tharreau

**Affiliations:** 1 CIRAD, UMR BGPITA A54/K, F 34398, Montpellier, France; 2 INRA, UMR BGPITA A54/K, F 34398, Montpellier, France

**Keywords:** center of origin, domestication, *Magnaporthe*, migration, population, rice

## Abstract

- Inferring invasion routes and identifying reservoirs of diversity of plant pathogens are essential in proposing new strategies for their control. *Magnaporthe oryzae,* the fungus responsible for rice blast disease, has invaded all rice growing areas. Virulent genotypes regularly (re)emerge, causing rapid resistance breakdowns. However, the world-wide genetic subdivision of *M. oryzae* populations on rice and its past history of invasion have never been elucidated.
- In order to investigate the centers of diversity, origin and migration of *M. oryzae* on rice, we analyzed the genetic diversity of 55 populations from 15 countries.
- Three genetic clusters were identified world-wide. Asia was the center of diversity and the origin of most migrations to other continents. In Asia, two centers of diversity were revealed in the Himalayan foothills: South China–Laos–North Thailand, and western Nepal. Sexual reproduction persisted only in the South China–Laos–North Thailand region, which was identified as the putative center of origin of all *M. oryzae* populations on rice.
- Our results suggest a scenario of early evolution of *M. oryzae* on rice that matches the past history of rice domestication. This study confirms that crop domestication may have considerable influence on the pestification process of natural enemies.

## Introduction

Increasing globalization of trade and climatic changes enhance the probability of (re-) emergence of invasive pests. These latter are usually difficult to manage without using pesticides, and represent unprecedented economic and food safety risks (Estoup & Guillemaud, 2010). Understanding how past events shaped the contemporary genetic diversity of pathogen populations helps in predicting their future change (Lawson Handley *et al.*, 2011). Pathogen species of domesticated organisms have likely adapted to their human-disturbed environments during the domestication process, enabling them to invade new habitats with similar characteristics (Lee & Gelembiuk, 2008; Hufbauer *et al.*, 2012). The homogenization of cultivated landscapes over different geographical areas has enhanced the invasion capacity of crop pathogens, by minimizing the magnitude of the evolutionary response required to adapt to new environments (Stukenbrock & McDonald, 2008; Estoup & Guillemaud, 2010; Guillemaud *et al.*, 2011). Plant pathogenic fungi, which represent major threats for several crops (Fisher *et al.*, 2012), are outstanding examples of pests whose evolutionary potential has been shaped by ‘Anthropogenically induced adaptation to invade’ (Hufbauer *et al.*, 2012). During the last decade, this has been exemplified for several emergent or re-emergent fungal pathogens (Banke & McDonald, 2005; Brunner *et al.*, 2007; Gomez-Alpizar *et al.*, 2007; Stukenbrock *et al.*, 2007; Gladieux *et al.*, 2008; Stukenbrock & McDonald, 2008).

*Magnaporthe oryzae* (*Mo*) is the Ascomycete fungus responsible for the most damaging rice disease world-wide: blast. This model species for the study of host–pathogen interactions (Valent, 1990; Dean *et al.*, 2012) is a major threat to food security (Pennisi, 2010). Disease control is mainly genetic, but complete resistance genes of rice varieties are frequently overcome following the emergence of virulent blast strains. The molecular mechanisms of virulence acquisition have been documented (Dai *et al.*, 2010; Takahashi *et al.*, 2010; Chuma *et al.*, 2011; Kanzaki *et al.*, 2012; Cesari *et al.*, 2013), but how such strains emerge and spread among and between populations remains misunderstood. Rice (*Oryza sativa*; *Os*), one of the host plants of *Mo*, probably originated from two independent domestication events involving wild rice *O. rufipogon c*. 7000 yr bp. These events occurred in two different regions that represent centers of diversification of cultivated rice, and resulted in two subspecies: *Os* ssp. *japonica* domesticated in southern China (Cheng *et al.*, 2003; Londo *et al.*, 2006; Fuller *et al.*, 2009; Huang *et al.*, 2012) and *Os* ssp. *indica* domesticated south of the Himalayas (likely eastern India, Myanmar or Thailand). However, the early history of *Mo* on cultivated rice, and especially the impact of rice domestication on *Mo* pestification, is still debated. How far human-mediated movements of infected materials are involved in inter-continental migration, especially between Asia and other continents, has seldom been investigated (Tharreau *et al.*, 2009). Previous studies on blast populations give fragmentary information regarding these key questions. A single origin of *Mo* on cultivated rice has been suggested due to a single acquisition of pathogenicity (Shull & Hamer, 1994), possibly following a host shift from strains attacking foxtail millets (*Setaria* spp), probably in South China *c*. 10 000 yr ago where both rice and foxtail millet were domesticated and co-cultivated (Couch *et al.*, 2005). While *Mo* reproduces asexually in most areas (Zeigler, 1998; Saleh *et al.*, 2012), the presence of recombining populations is suspected in northeastern India (Zeigler, 1998; Kumar *et al.*, 1999) and evidenced in Yunnan province, China (Saleh *et al.*, 2012). Because sexual reproduction is believed to be ancestral in species that reproduce both sexually and asexually (Schurko & Logsdon, 2008), this geographic distribution also supports the hypothesis of Asia as the center of origin of the species. Finally, numerous studies have described the genetic diversity of *Mo* in different parts of the world using various genetic markers. Reviewing such studies, Zeigler (1998) concluded that the genetic diversity of *Mo* was higher in the area encompassing South, East and Southeast Asia than in other regions. More than 50 clonal lineages per country were characterized in India (Kumar *et al.*, 1999), China (Chen *et al.*, 2006) and Thailand (Zeigler, 1998), and 2–10 lineages were detected in Japan, (Don *et al.*, 1999a), Korea (Park *et al.*, 2003, 2008), the Philippines (Chen *et al.*, 1995; Zeigler *et al.*, 1995) and Vietnam (Don *et al.*, 1999b). Outside of China, India and Thailand, by contrast, fewer (4–17) lineages were detected in Europe (Roumen *et al.*, 1997; Piotti *et al.*, 2005), Iran (Javan-Nikkah *et al.*, 2004), USA (Levy *et al.*, 1991; Xia *et al.*, 1993, 2000; Correll *et al.*, 2009), Argentina (Consolo *et al.*, 2008), Colombia (Levy *et al.*, 1993; Zeigler, 1998), Cuba (Fuentes *et al.*, 2003) and West Africa (Takan *et al.*, 2012). Besides these local studies, few attempts have been made to describe the population structure of *Mo* at a more global scale (Soubabère *et al*., 2000). In the most recent one, Tharreau *et al*. (2009) analyzed the genetic diversity of a world-wide collection of strains, and depicted a world-wide genetic structure of three clusters, Asian strains being scattered in the three clusters.

In sum these studies suggest that the origin of *Mo* strains pathogenic on rice may be in Asia and that most of the genetic diversity observed around the world is represented in this region. Thus, it is tempting to hypothesize that Asia may also be the center from which *Mo* dispersed towards the rest of the world. However, previous studies were limited to one country and were based on collections of strains maximizing diversity, not on population sampling. Testing these hypotheses requires population sampling covering native and secondary areas, coupled with analyses of population subdivision and genetic diversity without *a priori* on the population genetic structure. Such study – still lacking for *Mo* – is crucial to elucidate the routes and modalities of introduction, and would contribute to our understanding of how the pathogen emerged and spread, providing important clues for control methods to limit migrations of virulent strains and to improve the management of resistant varieties. Besides confirming the preliminary results of Tharreau *et al*. (2009) with appropriate population sampling and additional methods, the aim of the present work was therefore to address several questions about the origin, population structure and migration routes of world-wide populations of *Mo*. Using populations from different continents (Asia, Europe, the Americas and Africa), we asked: What are the main genetic groups in world-wide *Mo* populations? Can we localize one (or several) center(s) of genetic diversity? Based on the reproductive mode in the populations analyzed, can we infer the putative center of origin of the pathogen? Can we localize the geographic origin(s) of *Mo* migrations throughout the world?

## Materials and Methods

### Sampling

We used 55 world-wide population samples of *Mo* rice strains isolated between 2000 and 2009 (1372 strains in total; Table 1). A population was composed of strains collected in the same field on the same variety. In two cases (CH1 and MD1), different samples were collected at the same place but over two consecutive years; we grouped them as a single population after having verified that they were not genetically differentiated based on *F*_ST_ estimated from microsatellite markers. These 55 populations represented all continents (but without West African populations) with 423, 422, 136 and 391 strains from Asia, Europe, the Americas and Africa, respectively. Fungal strains were obtained after monospore isolation, as previously described by Silué & Nottéghem (1990) and stored as described by Valent *et al*. (1986).

**Table 1:** Geographic origin and basic information on genetic diversity of 55 population samples of *Magnaporthe oryzae*

Area	Country	Sample	*N*	*H*_*n.b*._	*N*_*a*_	*N*_*p*_	*MLG*	*G : N*	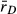
Asia	China	CH1	107	0.629	6.9	1.6	82	77%	0.069
–	–	CH2	38	0.522	3.4	0.2	21	55%	0.292
–	–	CH3	23	0.499	3.7	0.1	11	48%	0.189
–	–	CH4	25	0.051	1.3	0	4	16%	0.209
–	–	CH5	28	0.541	3.8	0	19	68%	0.094
–	–	CH6	30	0.253	2.6	0	7	23%	0.368
–	–	CH7	14	0.256	2.1	0	9	64%	0.150
–	Indonesia	ID1	20	0.277	2.3	0.1	10	50%	0.135
–	–	ID2	19	0.089	1.4	0.1	4	21%	0.095
–	–	ID3	16	0.114	1.7	0	6	38%	0.053
–	Laos	LA1	15	0.569	3.4	0.4	12	80%	0.154
–	–	LA2	9	0.518	3.5	0.2	8	89%	0.073
–	Nepal	NP1	31	0.168	2.6	0.1	11	35%	0.295
–	–	NP2	15	0.403	2.8	0.3	6	40%	0.330
–	–	NP3	6	0.491	2.4	0.1	4	67%	0.651
–	Thailand	TH	27	0.460	3.6	0.1	18	67%	0.166
Europe/MB	France	FR1	23	0.124	1.7	0	9	39%	0.050
–	–	FR2	17	0.163	1.9	0	9	53%	−0.010
–	–	FR3	18	0.112	1.6	0	6	33%	0.113
–	–	FR4	17	0.110	1.6	0	8	47%	0.009
–	–	FR5	22	0.019	1.2	0.1	3	14%	nd
–	–	FR6	37	0.317	2.3	0.1	8	22%	0.398
–	–	FR7	15	0.024	1.1	0	2	13%	nd
–	Greece	GR1	10	0.246	1.7	0	6	60%	0.261
–	–	GR2	10	0.183	1.8	0	6	60%	0.144
–	–	GR3	10	0.225	1.7	0	4	40%	0.456
–	–	GR4	9	0.102	1.5	0	3	33%	nd
–	–	GR5	9	0.167	1.6	0	3	33%	nd
–	–	GR6	10	0.190	2	0	4	40%	0.544
–	–	GR7	9	0.115	1.3	0	3	33%	nd
–	Hungary	HN1	7	0.070	1.2	0	3	43%	nd
–	–	HN2	3	0.053	1.1	0	2	67%	nd
–	–	HN3	7	0.101	1.3	0	4	57%	−0.055
–	Morocco	MC	15	0.169	1.9	0.1	8	53%	0.097
–	Spain	SP1	12	0.260	2	0	6	50%	0.344
–	–	SP2	31	0.204	2.1	0	11	35%	0.245
–	–	SP3	11	0.258	1.7	0	5	45%	0.545
–	–	SP4	22	0.278	2.1	0	9	41%	0.399
–	–	SP5	13	0.039	1.2	0	3	23%	nd
–	–	SP6	9	0.052	1.1	0	2	22%	nd
–	–	SP7	10	0.044	1.1	0	2	20%	nd
–	–	SP8	18	0.052	1.1	0	2	11%	nd
–	–	SP9	29	0.057	1.3	0.1	4	14%	−0.046
–	Turkey	TR	19	0.048	1.3	0	4	21%	−0.079
Americas	Colombia	CL1	17	0.083	1.4	0	2	12%	nd
–	–	CL2	31	0.052	1.4	0.1	5	16%	−0.057
–	French Guyana	GY	12	0.219	1.9	0.2	3	25%	nd
–	USA	USA1	37	0.558	3.2	0.1	14	38%	0.469
–	–	USA2	39	0.018	1.3	0	3	8%	0.280
Africa	Madagascar	MD1	264	0.039	3.7	0.3	24	9%	0.191
–	–	MD2	27	0.169	2	0	10	37%	0.047
–	–	MD3	37	0.115	2.2	0	12	32%	0.061
–	–	MD4	15	0.000	1	0	1	7%	nd
–	–	MD5	23	0.193	2.4	0	11	48%	0.022
–	–	MD6	25	0.074	1.7	0	7	28%	−0.007

The first three columns give the area of origin (MB, Mediterranean Basin), the country of origin, and the name of the samples. *N,* sample size; *H*_n.b_, unbiased gene diversity; *N*_a_, mean number of alleles per locus; *N*_p_, mean number of private alleles per locus; *G*, number of multilocus genotypes (*MLG*); *G : N*, proportion of unique MLG; 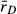, multilocus linkage disequilibrium (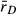 was not calculated (nd) when the number of *MLGs* was below 4).

### Determination of mating type and fertility

The mating type and female fertility of 600 strains were determined by *in vitro* crosses as described by Nottéghem & Silué (1992). Mating in *Mo* requires strains of opposite type and at least one of the strains must be female-fertile (able to produce perithecia). Crosses were performed by confronting the tested strain to female-fertile strains for which the mating type is known (reference strains). Mat1 reference strains were IN1, TH12, CH999 and CH1003. Mat2 reference strains were GY11, TH16, CH997 and CH1019. Tested strains were classified as Mat1 when inducing or forming perithecia with a Mat2 reference strain (and conversely). Tested strains were classified as female-fertile when forming perithecia with reference strains. For 175 additional strains, the mating type was determined by PCR amplification with the primers specific of Mat1 and of Mat2 (Xu & Hamer, 1995). In those cases, female fertility was not assessed.

### DNA extraction and microsatellites amplification

DNA extraction was performed following a CIAA procedure (Adreit *et al.*, 2007). All strains were genotyped with 10 microsatellites markers (Supporting Information Table S1) previously developed (Kaye *et al.*, 2003; Adreit *et al.*, 2007). Amplifications and allele size determination were performed as previously described (Saleh *et al.*, 2012).

### Indices of genetic diversity and linkage disequilibrium in populations

For each population, the mean number of alleles per locus *N*_a_, and the unbiased gene diversity *H*_n.b._ (Nei, 1987) were calculated using Genetix v4.05 (Belkhir *et al.*, 2004). We calculated the mean number of private alleles (*N*_p_) as the number of alleles that were present only in one population, averaged over the ten markers. The number of multilocus genotypes (MLG) and the index of association 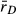 were calculated using Multilocus v1.3 (Agapow & Burt, 2001). The proportion of unique MLG in each population was calculated as the *G : N* ratio (*G*, number of MLG; *N*, sample size).

### Clustering and assignment analyses

Clustering methods were used to estimate the number of genetic groups that best explained the data. We used the Discriminant Analysis of Principal Components (DAPC; Jombart *et al.*, 2010) that does not require any assumption on the biology of the organism, especially regarding panmixia. The DAPC was conducted using the *adegenet* package (v1.3-1) for the R software (v2.13.1; Vienna, Austria). We used the K-means procedure implemented in the function *find.cluster* to infer *K,* the optimal number of clusters, and let *K* vary between 1 and 60. *K* was determined using the Bayesian Information Criterion (BIC): if the function BIC = *f*(*K*) was U-shaped, then *K* was the abscissa of the minimum of this function; otherwise it corresponds to the point where the BIC decay rate abruptly changed (*K* then being the value after which the change in BIC was negligible; Jombart *et al.*, 2010).

We also used the Structure Bayesian method (Pritchard *et al.*, 2000; Falush *et al.*, 2003). The basic assumption underlying this method is that the analyzed population can be theoretically subdivided into panmictic clusters. However, the method is supposed to be robust to departure from panmixia, and has given relevant results also in clonal or autogamous organisms (Garris *et al.*, 2005; Bahri *et al.*, 2009). We used the model with correlated allele frequencies and allowing admixture. Structure was run for *K* ranging from one to 32 with 10 replicates for each value of *K*. For each run, an 80 000-step Monte Carlo Markov Chain (MCMC) was performed after a 20 000 steps burn-in period. No *a priori* information was used on the assignments of individuals. We determined *K*_e_, the optimal number of clusters, according to Evanno *et al*. (2005). Results were also checked for *K*_e_−1 and *K*_e_* *+ 1. Individuals were assigned to a cluster if their probability of ancestry in this cluster was over the empirical cut-off of 0.7.

Unbiased gene diversity *H*_n.b_ and the mean number of private alleles *N*_p_ were assessed for each genetic cluster as described above.

### Genetic differentiation and genetic distances between populations or clusters

Pairwise *F*_ST_ (Weir & Cockerham, 1984) was calculated between clusters and the null hypothesis *F*_ST_ = 0 was tested using exact tests implemented in Genepop v4 (Raymond & Rousset, 1995). For the clusters inferred in Asia, the D_A_ chord genetic distance calculated between all pairs of clusters was used to build an unrooted neighbor-joining tree using Populations v1.2.3.1 (O. Langella, http://bioinformatics.org/∼tryphon/populations/). We visualized the number of alleles shared between Asian clusters using a Venn diagram (package *Venn.diagramm* of the R software). At the world-wide scale, we calculated the number of MLG shared between different countries. We verified that these MLGs were real clones by calculating *P*_sex_, the probability that a genotype arose in several individuals within a population by independent reproduction events (Parks & Werth, 1993; Tibayrenc *et al.*, 1990; Arnaud-Haond *et al.*, 2005) using MLGsim (Stenberg *et al.*, 2003). Identical MLGs with significantly low *P*_sex_ values may be considered as belonging to the same clonal lineage. The program performs simulations of populations under random mating to assess the significance of *P*_sex_ values.

An unrooted neighbor-joining tree of the world-wide populations was built from the pairwise D_A_ distance (when the sample size was higher than 6).

### Correlations between clusters assignments and biological features

Chi-squared tests were performed to assess if cluster assignment was independent from the rice subspecies (*indica* or *japonica*) the strains were collected on, the strain mating type (Mat1 and Mat2) and the female fertility status (female-fertile or female-sterile).

### Migration capacities of *M. oryzae* in Asia

We evaluated spatial autocorrelation at different spatial scales in Asia using SPAGeDi v1.3 (Hardy & Vekemans, 2002). We calculated Moran's index, *I*, for all pairs of Asian individuals (either globally or by genetic cluster), in different distances classes. *I* ranges between −1 (negative spatial autocorrelation) and 1 (positive spatial autocorrelation). Distances classes were manually selected, class limits being positioned at breakpoints in the range of pairwise distances. The significance of *I* values was assessed by performing 1000 permutations. Linear regressions of *I* against distance (or its logarithm), as well as the significance of the regression slopes, were also estimated; the intrapopulation classes (i.e. individuals with identical geographic coordinates) were not considered in these analyses.

## Results

### Genetic structure and genetic diversity of world-wide populations

In order to infer the centers of diversity of *Magnaporthe oryzae* (*Mo*), the first stage was to study the genetic structure over all populations. We first inferred population subdivision at the global scale and evaluated genetic differentiation between genetic groups using *F*_ST_. Then we studied the distribution of genetic diversity with regards to genetic structure and geography, within populations and within clusters.

We genotyped 1372 strains from 55 population samples of *Mo* rice strains from 15 countries (Table 1) with 10 microsatellite markers. In the DAPC, the 40 principal components retained explained more than 90% of the observed variance. The DAPC segregated the individuals into three genetic clusters (A, B, C). The Structure analysis also resulted in three clusters, the 10 replicates being 100% reproducible. The assignments of individuals to the three clusters were identical with DAPC and Structure (Fig. S1), except for four individuals (one from CH5, one from TH and two from SP1). With Structure, only 16 (among which 14 Asian strains) showed admixture signals (mixed ancestry in at least two clusters), but the DAPC assigned these 16 individuals to a single cluster.

The three clusters were highly differentiated (*F*_ST_ = 0.41, 0.44 and 0.65 between clusters A and B, A and C, and B and C, respectively) when compared to the average differentiation in phytopathogenic fungi (*F*_ST_ = 0.2 ± 0.05; Giraud *et al.*, 2008).

**Fig 1:**
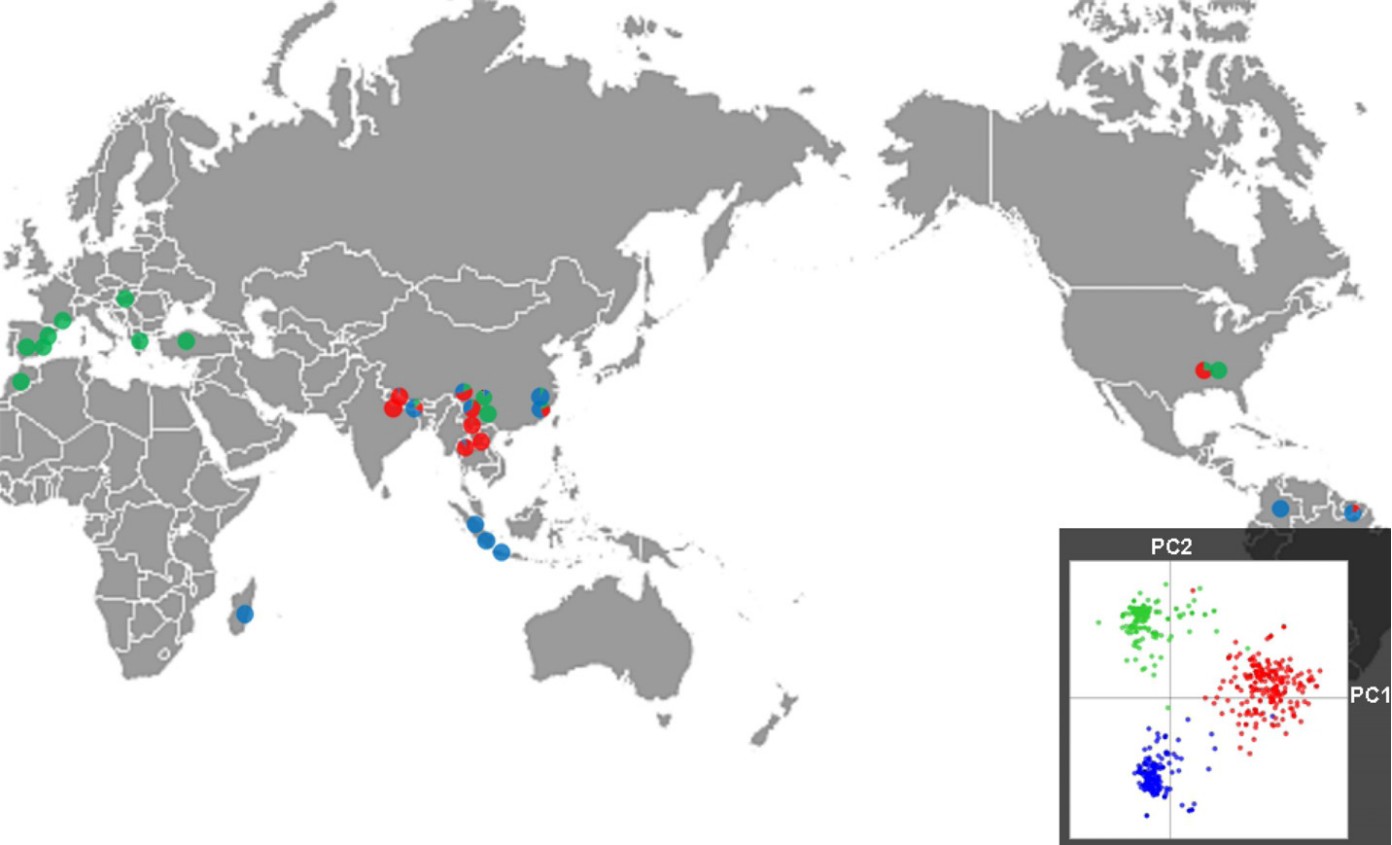
Proportion of strains belonging to the three clusters inferred using Discriminant Analysis of Principal Components (DAPC) in 55 world-wide samples of *Magnaporthe oryzae*. Cluster A, red; cluster B, green; cluster C, blue. The inset gives the coordinates of the individuals on the first two axes of the Principal Components Analysis.

The observed subdivision was highly associated with the geographic origin of the strains (Fig.1). All individuals from Europe/Mediterranean Basin belonged to cluster B and all individuals from Madagascar and Indonesia belonged to cluster C. Individuals from South America belonged to cluster C except for two Guyanese strains assigned to cluster A. Individuals from population USA1 were assigned to two clusters (A and B). Conversely, the three clusters described at the world-wide scale were all represented in Asia. Moreover, Asia was the only region in which the three clusters were represented in the same populations (CH2, CH3, CH5, CH6, NP2). Cluster A was over-represented in Asia (235 strains over 265).

We also looked at genetic diversity within clusters, within populations and within clusters × populations combinations.

Gene diversity (*H*_n.b._) calculated over all individuals within clusters, was at least two times higher in cluster A than in the other clusters (*H*_n.b._ = 0.68, 0.32 and 0.23 for clusters A, B and C, respectively). The mean number of private alleles (*N*_p_) was five times higher in cluster A compared to the other clusters (*N*_p_ = 5, 0.7 and 1 for clusters A, B and C, respectively).

**Fig 2:**
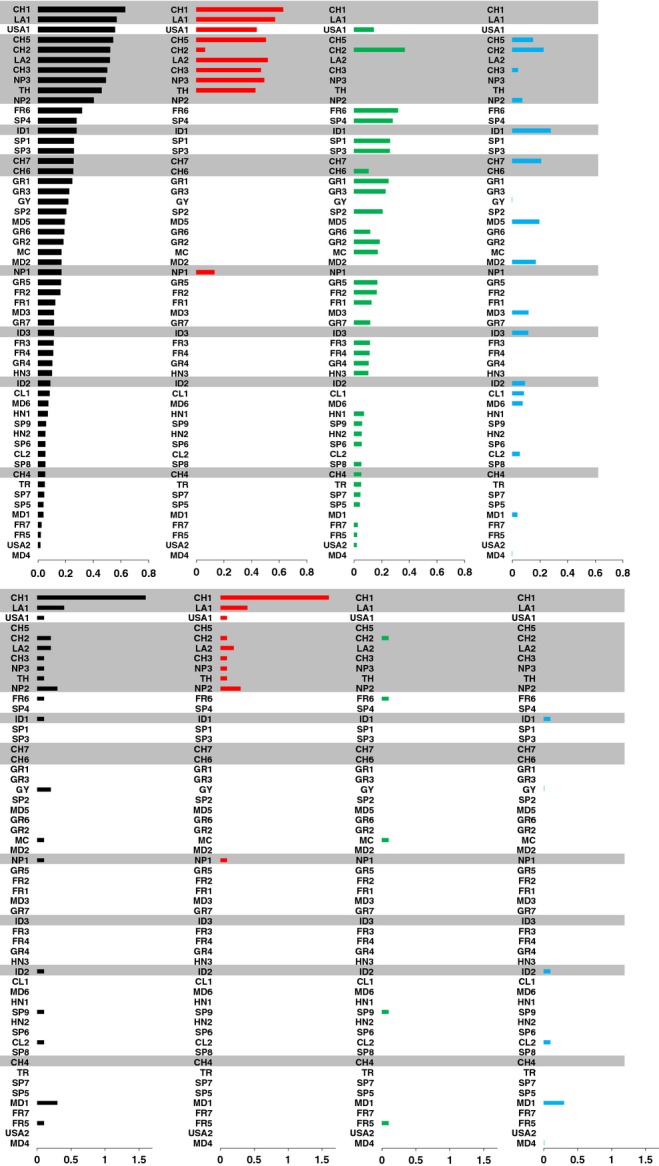
Measures of gene diversity (*H*_n.b_., upper panel) and mean number of private alleles (*N*_p_, lower panel) in samples (black bars) and in subsamples of *Magnaporthe oryzae* individuals from the same genetic cluster (colored bars). Population names are given in decreasing order of total *H*_n.b._. Red bars, subsample of individuals assigned to cluster A; green bars, subsample of individuals assigned to cluster B; blue bars, subsample of individuals assigned to cluster C. Asian samples and subsamples are shaded in gray. *H*_n.b._ and *N*_p_ were calculated only if the number of strains was higher than 6.

*H*_n.b._ and *N*_p_ were also calculated within each population (Fig.2, black bars). In populations from Asia the *H*_n.b._ mean value reached 0.38 ± 0.19 SD, whereas it was only 0.14 ± 0.09, 0.19 ± 0.22 and 0.10 ± 0.07 in populations from Europe/Mediterranean Basin, the Americas and Madagascar, respectively. Similarly, the mean *N*_p_ value was 0.19 ± 0.38 in Asia but only 0.01 ± 0.04, 0.08 ± 0.08 and 0.05 ± 0.12 in Europe/Mediterranean Basin, the Americas and Madagascar, respectively. The lowest mean values of *H*_n.b_ and *N*_p_ among Asian countries were found in Indonesia (*H*_n.b_ = 0.16 ± 0.10 and *N*_p_ = 0.07 ± 0.06). Because sample sizes were highly different between some populations, and because gene diversity is known to be dependent on sample size (Leberg, 2002), we randomly sampled ten individuals in each of the 55 populations for which sample size was higher than ten, and recalculated *H*_n.b._ and *N*_p_ in these 55 re-samplings (with five replicates of this re-sampling procedure). This confirmed that gene diversity was higher in Asian populations (Table S2).

Hence, the higher genetic diversity observed in Asian populations had two explanations: some Asian populations encompassed individuals from the three clusters (e.g. CH2 and CH5), and others mainly encompassed individuals from the most diverse cluster, A (e.g. CH1 and LA1). To make the difference between these two potential causes, within each population we grouped strains belonging to the same cluster and calculated *H*_n.b._ and *N*_p_ on these subsamples (Fig.2, colored bars). To get reliable values, this was performed only on subsamples containing more than six individuals. The subsamples presenting the highest values for *H*_n.b._ and *N*_p_ belonged to cluster A. Only one subsample from outside Asia (USA1) had a gene diversity comparable to Asian subsamples but its number of private alleles was much lower. The most diverse subsamples belonging to cluster B and C also originated from Asia.

Hence, Asia was the best candidate to be, or include, the center of diversity of *Mo* compared to the other continents. To localize this center more precisely, we analyzed the genetic structure and the distribution of genetic diversity inside Asia.

### Localization of the centers of diversity in Asia

At the Asian scale, we also inferred genetic structure using assignments methods. We evaluated *F*_ST_ between genetic groups. We then studied the distribution of genetic diversity with regards to genetic structure and geography: within populations and within clusters.

**Fig 3:**
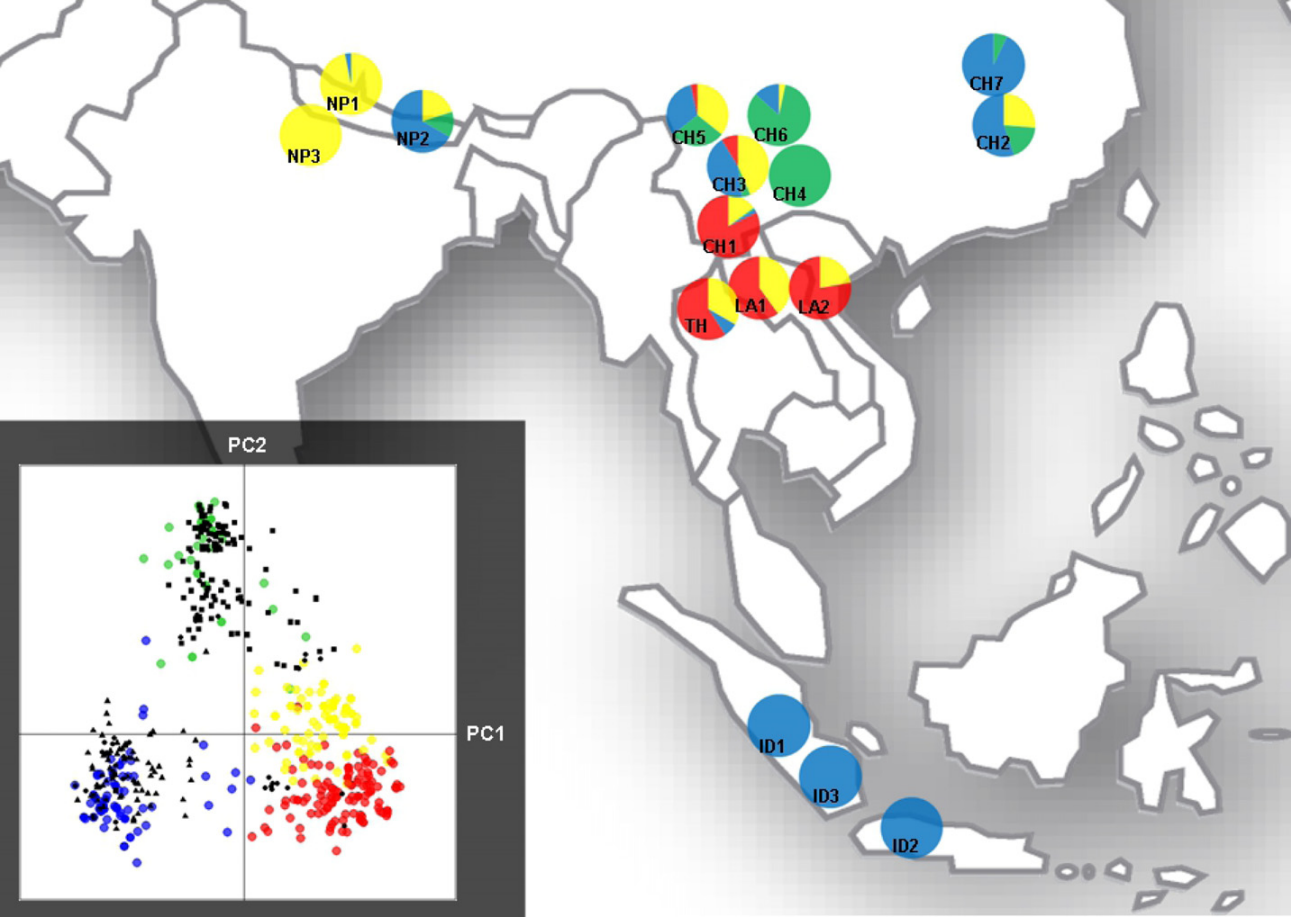
Discriminant Analysis of Principal Components (DAPC) on the 423 Asian strains of *Magnaporthe oryzae*. Cluster 1, yellow; cluster 2, green; cluster 3, blue; cluster 4, red. Samples are labelled according to their country of origin (CH, China; ID, Indonesia; LA, Laos; NP, Nepal; TH, Thailand). The inset gives the coordinates of the individuals on the first two axes of the Principal Components Analysis. Genotypes of strains from outside of Asia are represented in black: squares for Europe/Mediterranean basin, triangles for Madagascar, discs for Americas.

The DAPC performed on the 423 Asian individuals revealed that the region was organized in four genetic clusters (numbered from 1 to 4; Fig.3). The subdivision inferred using Structure was congruent with this result, and individual assignments to the four clusters were identical among the 10 replicates. 386 individuals could be assigned to a single cluster, the 37 remaining individuals showing admixture signal. The DAPC achieved to assign these 37 individuals to a single group. Individual assignments were identical with DAPC and with Structure, except for 12 individuals (six from CH1, three from LA1 and three from TH). Nine out of these 12 were assigned to cluster 1 by Structure and to cluster 4 by DAPC, or assigned to cluster 4 by Structure and to cluster 1 by DAPC.

**Fig 4:**
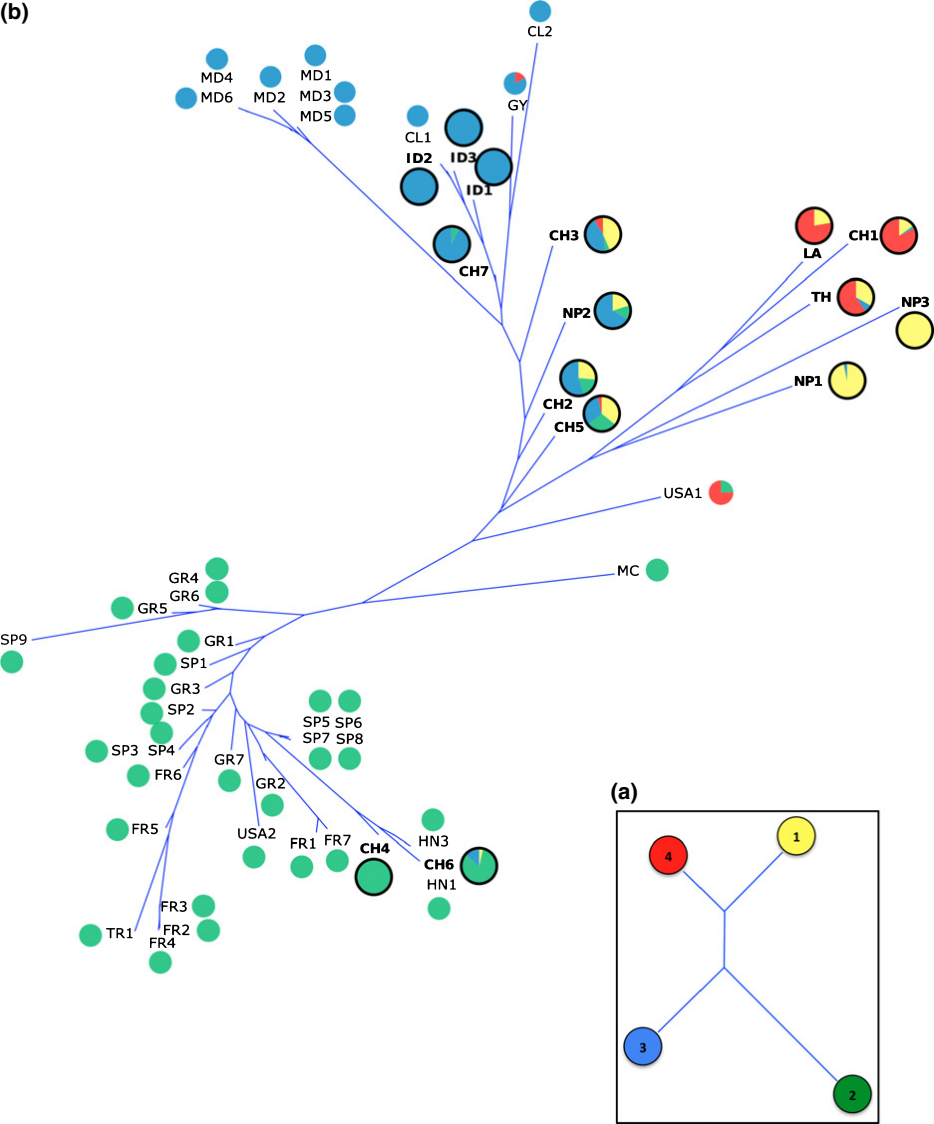
Unrooted neighbor-joining trees based on the D_A_ chord distance between *Magnaporthe oryzae* clusters or samples (a) between pairs of Asian clusters, and (b) between pairs of world-wide samples for which sample size was higher than 6.

Each cluster was significantly differentiated from the others: pairwise *F*_ST_ values were always higher than 0.2, the lowest *F*_ST_ being between clusters 1 and 4 (*F*_ST_ = 0.44 between clusters 1 and 2; 0.49 between clusters 1 and 3; 0.27 between clusters 1 and 4; 0.63 between clusters 2 and 3; 0.48 between clusters 2 and 4; and 0.38 between clusters 3 and 4; *P*-values of Fisher's exact tests as implemented in Genepop v4 were below 10^−5^ for all pair of clusters, allowing rejection of the null hypothesis of no differentiation). The pairwise D_A_ chord distance calculated among the four Asian clusters confirmed that clusters 1 and 4 were more closely related to each other than to clusters 2 and 3 (Fig.4a).

All strains assigned to clusters 1 and 4 but one were assigned to cluster A in the world-wide analysis. Identically, the Asian cluster 2 mostly overlapped with the world-wide cluster B, and the Asian cluster 3 mostly overlapped with the world-wide cluster C. Therefore, the three-cluster structure depicted in Asia through the world-wide analysis was in accordance with the four-cluster structure obtained through the analysis of the Asian dataset alone. In Asia, different combinations of clusters could be observed in one geographic area (Fig.3). The populations from Yunnan province (South China), Laos and Thailand (CH1, LA1, LA2 and TH) shared a similar structure with individuals assigned essentially to cluster 4, and to a lower extent to cluster 1. The genetic composition of the other populations from Yunnan (CH3, CH4, CH5 and CH6) varied from one another, but the four clusters were detected there. The samples from Hunan province, China (CH2 and CH7), and the Indonesian populations were composed mainly of individuals from cluster 3. The Nepalese populations encompassed mainly individuals from cluster 1 (NP1 and NP3) or from cluster 3 (NP2).

Consistent with the three-cluster structure at the global scale, gene diversity was higher in clusters 1 and 4 than in clusters 2 and 3 (*H*_n.b_: 0.50, 0.61, 0.26 and 0.26, respectively; Table 2). This also held for allelic diversity (*N*_a_: 7.9 and 7.7 in clusters 1 and 4, respectively; 3.6 and 5.5 in clusters 2 and 3, respectively), and for the mean number of private alleles per locus (*N*_p_: 1.9 and 1.4 in clusters 1 and 4, respectively; 0.4 and 0.3 in clusters 2 and 3, respectively). Hence, clusters 1 and 4 were more genetically diverse than clusters 2 and 3.

**Table 2:** Genetic diversity within each of the four clusters of *Magnaporthe oryzae* Asian strains inferred using Discriminant Analysis of Principal Components (DAPC)

Cluster	*N*	*H*_n.b_	*N*_a_	*N*_p_	*MLG*	*G : N*	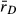
1	103	0.504	7.9	1.9	55	53%	0.097
2	72	0.256	3.6	0. 4	21	29%	0.182
3	131	0.259	5.5	0. 3	49	37%	0.078
4	124	0.610	7.7	1.4	96	77%	0.030

Number of individuals (*N*), unbiased gene diversity (*H*_n.b._), mean number of alleles per locus (*N*_a_), mean number of private alleles per locus (*N*_p_), number of multilocus genotypes (*MLG*), clonal richness (*G : N*), and multilocus linkage disequilibrium (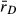).

The only difference between genetic subdivision at the worldwide and Asian scale was the split of world-wide cluster A into Asian clusters 1 and 4. Therefore, the calculation of gene diversity and mean number of private alleles in the clusters × populations combination at the Asian scale (Table S3), gave results similar to those obtained at the global scale (Fig.2, colored bars). Populations exhibiting the highest genetic diversity were those composed of individuals mainly assigned to cluster 1 and/or 4.

Two geographic areas were identified to be composed of populations showing this feature: a first region comprising South China (Yunnan, CH1), Laos (LA1, LA2) and Thailand (TH) where cluster 4 dominated, and a second region in Western Nepal (NP1 and NP3) where cluster 1 dominated. So, these results allowed us to define these two regions as two centers of diversity.

### Localization of the center of origin

We then wondered if one of these centers of diversity could correspond to the center of origin of *Mo* populations pathogenic on rice.

In order to test whether clusters 2 and 3 could originate from clusters 1 or 4, we compared the number of shared alleles between clusters (Fig. S2). We reasoned that any derived cluster should share more alleles with its cluster of origin than with other clusters. The number of shared alleles between clusters 2 and 1 was similar to the number shared between 2 and 4 (27 and 28, respectively). The number of shared alleles between clusters 3 and 1 was identical to the number shared between 3 and 4 (29). Thus, following this method, we could not figure out if clusters 2 and 3 are derived from cluster 1 or 4.

In organisms that can reproduce both sexually and asexually, the ability to reproduce sexually is believed to be an ancestral state that can be lost in certain conditions. In this case, sexual reproduction is expected in the center of origin rather than in introduced areas (Leslie & Klein, 1996). So, we looked for genetic and biological evidence of sexual reproduction in the four Asian clusters.

Footprints of recombination accompanying potential sexual reproduction were searched by measuring *G : N*, the proportion of multilocus genotypes (MLG) discriminated, and 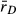, the multilocus linkage disequilibrium. In Asia, the average *G : N* in populations was 54% ± 23% SD (China: 50% ± 23%; Indonesia: 36% ± 15%; Laos: 84% ± 6%; Nepal: 57% ± 24% and Thailand: 67%; Table 1). The mean value of 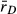 was 0.21 ± 0.15 SD (China: 0.20 ± 0.11; Indonesia: 0.09 ± 0.04; Laos: 0.11 ± 0.06; Nepal: 0.43 ± 0.20 and Thailand: 0.17; Table 1). The proportion of unique MLG was the highest in cluster 4 (*G : N*, 77%), intermediate in cluster 1 (*G : N*, 53%) and lowest in clusters 2 and 3 (*G : N*, 29% and 37%, respectively; Table 2). Multilocus linkage disequilibrium was lowest in cluster 4 (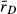: 0.030), intermediate in clusters 1 and 3 (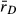: 0.097 and 0.078, respectively) and highest in cluster 2 (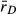: 0.182; Table 2).

Biological evidence of sexual reproduction was searched for by measuring the proportions of both mating types and of female-fertile strains within populations, both being required for sexual reproduction. Overall in Asia, the distribution of mating types (χ^2^ = 330, *P* = 3.2 × 10^−71^, df = 3) and of female-fertile strains (χ^2^ = 294, *P* = 2.0 × 10^−64^, df = 3) significantly depended on the cluster of origin. The frequency of Mat1 and Mat2 strains in clusters 1 and 4 was relatively balanced and not significantly different from a random distribution (Table 3). On the contrary, Mat1 strains were over-represented in cluster 2 and Mat2 strains were over-represented in cluster 3 (Table 3). Similarly, the proportion of female-fertile strains was highly different within the four clusters. Interestingly, the percentage of female-fertile strains was highest in clusters 4 (76%), intermediate in cluster 1 (42%), and lowest in clusters 2 and 3 (4% and 11%, respectively).

**Table 3:** Distribution of *Magnaporthe oryzae* individuals in genetic clusters at different scales as a function of the mating type and female fertility

	Mat1	Mat2			Female-fertile	Female-sterile	
(a) Asian scale							
1	36 (34.1)	33 (34.9)	69	1	27 (27.0)	38 (38.0)	65
2	61 (34.1)	8 (34.9)	69	2	3 (28.6)	66 (40.4)	69
3	22 (38.0)	55 (39.0)	77	3	5 (18.7)	40 (26.3)	45
4	44 (56.8)	71 (58.2)	115	4	87 (47.7)	28 (67.3)	115
	163	167	330		122	172	294
(b) Global scale							
A	92 (88.7)	132 (135.3)	224	A	116 (46.5)	111 (180.5)	227
B	195 (80.0)	7 (122.0)	202	B	3 (33.0)	158 (128.0)	161
C	20 (138.3)	329 (210.8)	349	C	4 (43.5)	208 (168.5)	212
	307	468	775		123	477	600

Values in brackets are expected values for an independent assortment calculated on the overall frequencies of the different characters.

Altogether, the low genotypic diversity, the high linkage disequilibrium, the dominance of one mating type and the very low proportion of female-fertile strains in clusters 2 and 3 suggested a low probability that sexual reproduction occurred in these groups. Although Cluster 1 presented a higher genotypic diversity and balanced proportions of Mat1 and Mat2 strains, the average proportion of female-fertile strains and the high linkage disequilibrium were not in agreement with the expectations of sexual reproduction. However, the high genotypic diversity, the low linkage disequilibrium, the balanced proportions of Mat1 and Mat2 strains and the high proportions of female-fertile strains in cluster 4 were consistent with footprints of sexual reproduction in this genetic group. Hence, the region where cluster 4 dominates – that is, the region comprising South China (Yunnan), Laos and North Thailand – should be considered as a putative center of origin of *Mo* populations pathogenic on rice.

### Localizations of the centers of migration

All clusters are present together only in Asia, and hence we hypothesized that Asia could be the center of origin of world-wide migrations. To test this hypothesis, we wondered if strains from populations outside Asia could be genetically related to the Asian clusters. We calculated the coordinates of the 949 nonAsian strains as supplementary individuals in the DAPC performed on the 423 Asian strains using the function *pred.sup* (package *Adegenet*, R software). All European/Mediterranean strains but one were assigned to cluster 2 (inset of Fig.3). All South American and the Madagascan strains were assigned to cluster 3. The North American strains were assigned to clusters 1, 2 and 4. These results show that all strains from outside Asia can be related to one of the Asian genetic groups. This points to Asia as the center of dispersion of *Mo*. We further tested this hypothesis by analyzing the shared MLG between different countries.

**Fig 5:**
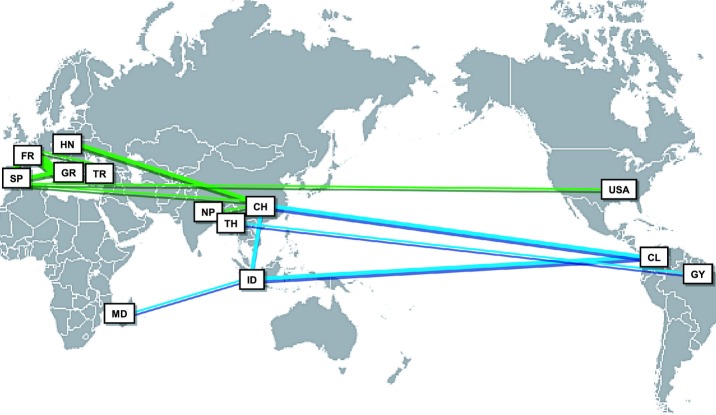
*Magnaporthe oryzae* multilocus genotypes (MLG) shared between countries. The width of the line is proportional to the number of shared MLG. Green lines and blue lines represent MLG that belong to cluster B and cluster C, respectively. CH, China; CL, Columbia; FR, France; GR, Greece; GY, French Guyana; HN, Hungary; ID, Indonesia; MD, Madagascar; NP, Nepal; SP, Spain; TH, Thailand; TR, Turkey; USA, United States of America.

Twenty MLG were shared between different populations within and between countries. Shared MLG between countries belonged to cluster B and to cluster C (Fig.5). No MLG were shared between countries within cluster A. Most of the shared MLG were found between countries of the same region: within Europe (five between France and Spain, five between France and Greece, two between Spain and Greece, one between France and Turkey) and within Asia (two between China and Indonesia, two between China and Nepal). Interestingly, several MLG were also shared between geographically distant countries, and especially between Asian countries and nonAsian ones (one between China and Spain, two between China and Hungary, two between China and Colombia, one between Indonesia and Madagascar, three between Indonesia and Colombia, one between Thailand and French Guyana). Only two MLG were shared between countries of different regions outside Asia: between Spain and USA. To validate these results, we tested the resolution power of the markers to discriminate clones, that can be affected in clonal organisms (Arnaud-Haond *et al.*, 2005, 2007). For MLGs that were both shared between at least two different populations and repeated within each population, we calculated *P*_sex_ within each population (the probability that repeated genotypes originate from distinct reproductive events). All the *P*_sex_ values were highly significant (Table S4), indicating that all the MLG shared between countries were real clones.

These results suggest long intercontinental migrations from Asia towards the other regions of the world, as well as intracontinental migrations. Furthermore, none of the MLG shared between continents were assigned to cluster A. In addition, only few strains assigned to cluster A were found out of Asia. This suggests that most of these migrations did not originate from the most ancestral genotypes, belonging to cluster A, but from more recent genotypes belonging to clusters B and C.

**Fig 6:**
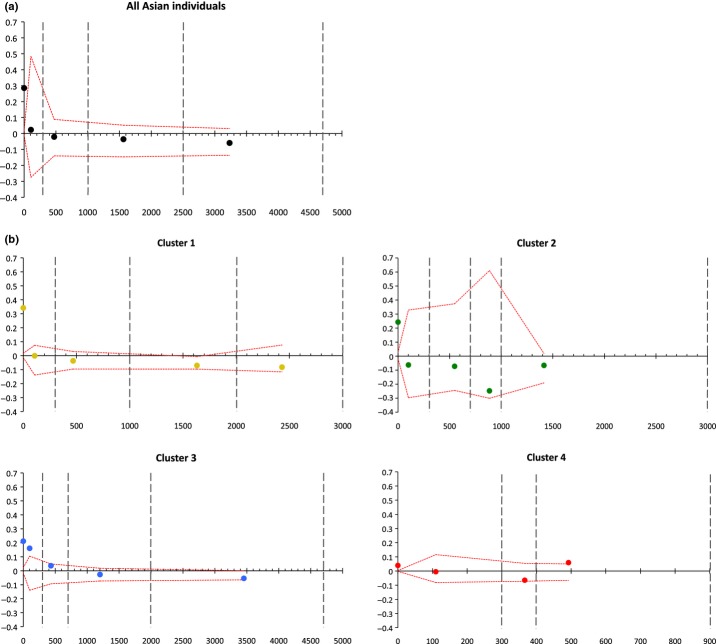
Spatial autocorrelation between *Magnaporthe oryzae* individuals in Asia. Moran's index *I* was calculated between pairs of Asian individuals for different distance classes, (a) for all Asian individuals; (b) in each of the four Asian genetic clusters. Abscissas of points are calculated as the mean pairwise distance of all pairs of individuals in the class considered. The points situated on abscissa 0 correspond to intrapopulation classes (individuals from the same spatial location). The red dotted lines represent upper and lower bounds of the 95% confidence interval, assessed after 1000 permutations. Vertical gray lines represent upper bounds of distance classes, manually chosen after the distribution of pairwise geographic distances.

In order to further address the migration capacities of *Mo* in Asia, we evaluated the spatial autocorrelation in this region using Moran's index *I*. When considering all Asian individuals (Fig.6a), significant positive spatial autocorrelation was observed only for the intrapopulation class (i.e. between pairs of individuals from the same spatial location). For all non-null distance classes, *I* was never significantly different from 0, indicating a random spatial pattern whatever the geographic scale considered. We obtained similar results when separating individuals by genetic cluster (Fig.6b), except for cluster C in which a weak but significantly positive spatial auto-correlation was observed in the distance class (0–300 km) (*I* = 0.16, *P* = 0.045, one-sided test). We never found any significant linear regression of *I* against distance (or its logarithm).

### Genetic distance between populations

At the world-wide scale, the neighbor-joining tree based on D_A_ chord distance between pairs of populations (except the HN2 sample which size was considered too small) showed that populations were grouped according to their mosaic composition in the different genetic clusters (Fig.4b). One clade grouped Asian LA, TH, CH1, NP1 and NP3 populations from the two centers of diversity (the first three being also from the putative center of origin). Another clade grouped Asian populations CH2, CH3, CH5 and NP2 which have a similar mosaic composition, with populations mostly or completely composed of individuals assigned to the Asian cluster 3 (CH7 and ID populations) or to world-wide cluster C (GY, CL and MD populations). The third clade grouped populations mainly composed of individuals belonging to Asian cluster 2 (CH4 and CH6) and to world-wide cluster B (populations from Europe/Mediterranean Basin).

## Discussion

Phylogeographic studies on different phytopathogenic fungi have shown a variety of situations regarding the co-localization of centers of origin, diversity and migration (Robert *et al.*, 2012). Here, we provided evidence that the center of origin of *Mo* on cultivated rice colocalizes with one of the two centers of diversity in South-East Asia but not with the center of dispersal towards the rest of the world.

### The centers of diversity of *Mo* match the centers of domestication of rice in Asia

The highest genetic diversity of *Mo* was found in Asia, at the regional scale (whole South-East Asia) as well as at the population scale, except for Indonesian populations. Allelic and gene diversities, and the number of private alleles were (respectively) two, three and four times higher in Asian populations than in populations from Europe/Mediterranean Basin, the Americas or Madagascar. Asian strains formed four genetic clusters that did not strictly match a single Asian country or region. Rather, Asian populations were composed of ‘mosaics’ of these clusters, whose composition corresponded roughly to the geography. Such a mosaic structure, described for example for the fungus *Venturia inequalis* in its area of origin (Gladieux *et al.*, 2008), shows that regional groups have a common but complex evolutionary origin involving mixing between several pre-existing genetic groups. Here, some populations were mostly composed of one cluster, and others comprised several clusters. The geographic distribution of the two most diverse clusters (1 and 4) determined two centers of diversity, one covering Yunnan province (China), Laos and Thailand, and the other located in western Nepal. Interestingly, these two centers of diversity match the putative domestication areas of rice, localized in South China and northeastern India (Londo *et al.*, 2006; Huang *et al.*, 2012).

### The center of origin of *Mo* matches one of its centers of diversity

Assuming that the ancestral reproductive character is sexuality rather than clonality (Schurko & Logsdon, 2008) and that asexual fungal crop pathogens might still reproduce sexually near their center of origin (Leslie & Klein, 1996), we inferred the center of origin of *Mo* by localizing those areas where footprints of sexual reproduction are detected. In *Mo*, sexual reproduction was previously inferred in India (Kumar *et al.*, 1999) and evidenced in South China (Saleh *et al.*, 2012). Here, genetic and biological evidences of past or present sexual reproduction designate the region comprising South China (Yunnan), Laos and North Thailand as the putative center of origin of *Mo* strains pathogenic on rice. Following Huang *et al*. (2012), this area matches the initial center of domestication of rice *Os* var. *japonica*. This result agrees with the formerly proposed hypothesis of a single origin of *Mo* strains pathogenic to cultivated rice in China following a host shift (Shull & Hamer, 1994; Couch *et al.*, 2005). As for other plant pathogens, the center of origin of *Mo* rice strains corresponds to one center of domestication of its host.

### Bridgehead effect in Asia: intercontinental migrations of *Mo* originated from secondary Asian areas

Tharreau *et al*. (2009) suggested the occurrence of intercontinental migrations of *Mo*. Our results confirmed that all secondary areas outside Asia actually had an Asian origin (Figs 5). At the global scale we found three clusters consistent with the four clusters found in Asia. The most diverse cluster (A) did not disperse much outside Asia, whereas the two others (B and C) were found extensively world-wide. Genetic diversity, especially the number of alleles shared between clusters, indicated that world-wide clusters B and C originated from Asian clusters 2 and 3, respectively. This illustrates a bridgehead effect in Asia; that is, that the secondary sources of long-range migrations are different from the centers of diversity and from the center of origin of blast (Lombaert *et al.*, 2010).

Our results also illustrate different invasion histories of secondary areas. In the European/Mediterranean Basin populations, all individuals but one belonged to a single world-wide cluster (B). Interestingly, the 69 Asian individuals also assigned to this cluster came mostly from two Chinese populations: 25 from CH4 and 26 from CH6. Moreover, we found common MLG between Hungarian strains and strains from CH3 and CH4 populations. There were also common MLG between Spanish strains and CH4 strains. Therefore, Yunnan, where CH3, CH4 and CH6 populations were collected, could likely represent the source of European/Mediterranean populations. A single introduction in the European/Mediterranean area is supported by the low genetic diversity observed in this region and by the fact that only one mating type (Mat1) is present there. The fungus was probably subsequently dispersed throughout Europe from a single, still undetermined entry.

Indonesian and Chinese populations CH2 and CH7 had common MLGs, showing possible exchange between the two regions. Madagascan populations had a single origin and belonged to the same cluster as the Chinese population CH7 and Indonesian populations. In addition, a common MLG was detected between Madagascar and Indonesia. So, either the Indonesian and Madagascan populations originated from the same genetic pool independently, or the Madagascan strains migrated from Indonesia. We favour the second hypothesis because the diversity observed in Madagascar is lower than in Indonesia, and because it matches the history of human migrations in these areas. The first human groups arrived in Madagascar from Indonesia at least 2000 yr ago (Hurles *et al.*, 2005), and may have carried with them blast-infected rice seeds.

Populations from Colombia and French Guyana belonged to the same cluster (C), and shared common MLGs with Asian populations: CL1 with CH2 and CH7 from Hunan province (China) and with ID1 and ID2 from Indonesia, and GY (especially the most common MLG of this population) with the Thailand population. So, *Mo* strains from South America may have different origins: Colombian populations might have originated either directly from western China or from Indonesia. In French Guyana, strains might have been introduced from Thailand or Vietnam, probably through recent migrations of H'Mongs.

The two North American populations obviously had different origins. All USA2 strains belonged to cluster B, like European strains, with two MLG shared with Spanish strains. So, this population might have originated either from the same Chinese genetic pool that migrated towards Europe, or directly from Europe itself. The USA1 population likely resulted from multiple introductions from different gene pools because it gathered strains belonging to two clusters (A and B): this suggests either two introductions from Asia, or one from Asia and one from Europe. Similar patterns of multiple introductions have already been described for other phytopathogenic fungi (Dutech *et al.*, 2010; Montarry *et al.*, 2010; González-Varela *et al.*, 2011; Robert *et al.*, 2012).

The world-wide organization of genetic diversity agrees with stochastic human-driven migrations outside Asia. Inside Asia, spatial autocorrelation analyses did not reveal any significant deviation from random spatial pattern. Hence, inside Asia, whatever the distance range considered (even at the intraregional geographic scale, i.e. up to 300 km), and whatever the genetic origin of individuals, migration events were also probably purely stochastic, and closely linked to movements of human groups or to seed exchanges. This agrees with previous knowledge on the short-distance natural migration capacities of *M. oryzae* (a few meters; D. Tharreau & J. L. Nottéghem, pers. comm.).

### Selection by the host might explain the differentiation of the secondary Asian centers

Our results support the hypothesis, also proposed by Levy *et al*. (1991) and Zeigler (1998), that selection by rice contributed to shaping the genetic structure of *Mo* populations. The higher diversity observed in clusters 1 and 4 may be explained by the higher diversity of the host in the Asian areas where clusters 1 and 4 are found. Indeed, both clusters encompassed mainly (179/226) strains collected on rice grown in upland conditions, where many traditional and diverse varieties were maintained (at least in the Asian regions sampled). Either the host diversity maintained directly pathogen genotypic diversity by selection, or indirectly by maintaining sexual reproduction.

We also found that membership to a particular genetic cluster was significantly associated with the prevalence of varieties of *indica* or *japonica* type in the area sampled. In Asia, cluster 3 was significantly over-represented in regions where *indica* rice is prevalent (Table S5a), and conversely all individuals but one from cluster 4 were sampled in regions were *japonica* rice is prevalent. World-wide, cluster B is prevalent in areas where *japonica* varieties – especially temperate – are grown (Table S5b), while cluster C is over-represented in areas where *indica* varieties are grown. Pathogenic specialization of blast on the different rice subspecies, suggested by Bonman *et al*. (1990), could explain this distribution. This specialization, that may not be strict and remains to be demonstrated for the clusters we identified, could be the result of host-pathogen coevolution. Indeed, *indica* and *japonica* rice subspecies were domesticated independently in two Asian areas, and our results show that population subdivision of blast in Asia matches this domestication process. Thus, following rice domestication, *Mo* possibly adapted independently to these two subspecies leading to differentiation in two clusters (B and C). This subdivision was maintained when these clusters spread into different countries because *Mo* was probably introduced with the rice varieties it was adapted to, and because different rice subspecies are used in different agrosystems, limiting the possibility of cross-adaptation between strains of the B cluster on *indica* rice varieties and of A cluster on *japonica*.

Altogether, our results suggest that *Mo* could have evolved as a major pathogen on cultivated rice through a host-tracking process, following a host shift from an unknown plant towards wild rice. Host-tracking – that is, the coevolution of the host and the pathogen during domestication (Stukenbrock & McDonald, 2008) – implies that both partners have the same center of origin, and has been suggested as an emerging mechanism for several important pathogenic fungi (Banke & McDonald, 2005; Gomez-Alpizar *et al.*, 2007; Raboin *et al.*, 2007; Gladieux *et al.*, 2008; Robert *et al.*, 2012).

### Sexual reproduction was probably lost during the differentiation of secondary Asian centers and intercontinental migrations

This study supports the hypothesis suggested by Levy *et al*. (1991) and Zeigler (1998) that *Mo* populations outside Asia derived recently from a limited set of founders. In addition, we found a nonrandom distribution of mating types and of female-fertile strains in the different clusters (χ^2^ = 441, *P* = 1.7 × 10^−96^, df = 2 and χ^2^ = 600, *P* = 5.1 × 10^−131^, df = 3, respectively; Table 3b), confirming the clonal structure of all nonAsian populations already demonstrated by Saleh *et al*. (2012). Within cluster A, we observed balanced proportions of the two mating types, and of female-fertile/female-sterile strains. Cluster B gathered almost only Mat1 strains (175/180), and cluster C almost only Mat2 strains (280/293). Cluster A gathered 94% of the total number of female-fertile strains, these strains being rare both within the two other clusters (2/97 in cluster B, 2/173 in cluster C). Furthermore our results suggest that the clonal populations found outside Asia likely originated from clonal Asian populations that pre-existed before world-wide migrations. Indeed, in Asian groups 2 and 3 mating type ratio is biased towards Mat1 and Mat2, respectively. Moreover, the frequency of female-fertile strains is low in these groups (4% and 11%, respectively). So, sexual reproduction was probably lost in these groups compared to groups 1 and 4 from which they likely derived. Following our ‘out of Asia’ dispersal scenario, the genetic groups B and C likely originated from groups 2 and 3, respectively. The absence of sexual reproduction in all areas outside Asia may thus be explained by migrations from source populations that were already exclusively clonal.

### Conclusion

Our study provides new insights on the native areas, diversity reservoirs and invasion routes of rice blast. We showed that several independent events of intercontinental migrations occurred which are likely linked with the transportation of infected materials. In a context of intense global exchanges, knowledge about these past events should lead to increased vigilance on the risk of introductions of new genotypes of the pathogen through the exchanges of rice seeds. Our work also exemplifies the role of plant domestication in shaping the population structure of plant pathogens. For the *Mo*/rice pathosystem, the independent domestication of *indica* and *japonica* rice subgroups led to the appearance of two genetic groups of the pathogen. Such a structure, probably accompanied by a specialization of the pathogen on the different rice subspecies, could be exploited to develop new strategies of deployment of resistance genes. Wild species have been proposed as a source of resistance genes to improve related crop species (Izawa & Shimamoto, 1996; Brar & Khush, 1997). A similar strategy could be used in the case of subspecies. It is likely that some so called major resistance genes, and defense mechanisms involving several genes, are specific for each rice subspecies. By introducing these genes in the other subspecies, the pathogen population would then be confronted by genes that it had never met before.

## Supporting Information

Fig S1 Genetic structure of worldwide individuals of *Magnaporthe oryzae*: comparison between the DAPC and Structure methods. **Fig. S2** Number of shared and specific alleles calculated over 10 microsatellites for the four *Magnaporthe oryzae* clusters identified in Asia.

Table S1 Characteristics of the 10 microsatellites used for *Magnaporthe oryzae* genotyping **Table S2** Unbiased gene diversity and number of private alleles in random subsamples performed in 46 *Magnaporthe oryzae* worldwide populations **Table S3** Gene diversity and mean number of private alleles in subsamples of *Magnaporthe oryzae* individuals from the same genetic cluster in Asia **Table S4** Information on *Magnaporthe oryzae* multilocus genotypes repeated within populations and shared between populations **Table S5** Distribution of *Magnaporthe oryzae* individuals in clusters according to the type of rice culture

## References

[b1] Adreit H, Santoso  , Andriantsimialona D, Utami DW, Nottéghem JL, Lebrun MH, Tharreau D (2007). Microsatellite markers for population studies of the rice blast fungus, *Magnaporthe grisea*. Molecular Ecology Notes.

[b2] Agapow PM, Burt A (2001). Indices of multilocus linkage disequilibrium. Molecular Ecology Notes.

[b3] Arnaud-Haond S, Alberto F, Teixeira S, Procaccini G, Serrao EA, Duarte CM (2005). Assessing genetic diversity in clonal organisms: low diversity or low resolution? Combining power and cost efficiency in selecting markers. Journal of Heredity.

[b4] Arnaud-Haond S, Duarte CM, Alberto F, Serrao EA (2007). Standardizing methods to address clonality in population studies. Molecular Ecology.

[b5] Bahri B, Leconte M, Ouffroukh A, De Vallavieille-Pope C, Enjalbert J (2009). Geographic limits of a clonal population of wheat yellow rust in the Mediterranean region. Molecular Ecology.

[b6] Banke S, McDonald BA (2005). Migration patterns among global populations of the pathogenic fungus *Mycosphaerella graminicola*. Molecular Ecology.

[b7] Belkhir K, Borsa P, Chikhi L, Raufaste N, Bonhomme F (1996–2004). GENETIX 4.05, logiciel sous Windows TM pour la génétique des populations.

[b8] Bonman JM, Mew TW, Koganezawa H, Vergel de Dios-Mew TI, Vera-Cruz CM, Medella ES, Kim KK, Nottéghem JL, Glaszmann JC (1990). Resistance to key diseases in sub-specific groups of rice. Phytopathology.

[b9] Brar DS, Khush GS (1997). Alien introgression in rice. Plant Molecular Biology.

[b10] Brunner PC, Schuerch S, McDonald BA (2007). The origin and colonization history of the barley scald pathogen *Rhynchosporium secalis*. Journal of Evolutionary Biology.

[b11] Cesari S, Thilliez G, Ribot C, Chalvon V, Michel C, Jauneau A, Rivas S, Alaux L, Kanzaki H, Okuyama Y (2013). The rice resistance protein pair RGA4/RGA5 recognizes the *Magnaporthe oryzae* effectors AVR-Pia and AVR1-CO39 by direct binding. Plant Cell.

[b12] Chen DH, Zeigler RS, Leung H, Nelson RJ (1995). Population structure of *Pyricularia grisea* at two screening sites in the Philippines. Phytopathology.

[b13] Chen QH, Wang YC, Zheng XB (2006). Genetic diversity of *Magnaporthe grisea* in China as revealed by DNA fingerprint haplotypes and pathotypes. Journal of Phytopathology.

[b14] Cheng CY, Motohashi R, Tsuchimoto S, Fukuta Y, Ohtsubo H, Ohtsubo E (2003). Polyphyletic origin of cultivated rice: based on the interspersion pattern of SINEs. Molecular Biology and Evolution.

[b15] Chuma I, Isobe C, Hotta Y, Ibaragi K, Futamata N, Kusaba M, Yoshida K, Terauchi R, Fujita Y, Nakayashiki H (2011). Multiple translocation of the AVR-Pita effector gene among chromosomes of the rice blast fungus *Magnaporthe oryzae* and related species. PLoS Pathogens.

[b16] Consolo VF, Cordo CA, Salerno GL (2008). DNA fingerprint and pathotype diversity of *Pyricularia oryzae* populations from Argentina. Australasian Plant Pathology.

[b17] Correll JC, Boza EJ, Seyran E, Cartwright RD, Jia Y, Lee FN, Wang GL, Valent B (2009). Examination of the rice blast pathogen population diversity in Arkansas, USA – Stable or unstable?. Advances in genetics, genomics and control of rice blast disease.

[b18] Couch BC, Fudal I, Lebrun MH, Tharreau D, Valent B, van Kim P, Nottéghem JL, Kohn L (2005). Origins of host-specific populations of the blast pathogen *Magnaporthe oryzae* in crop domestication with subsequent expansion of pandemic clones on rice and weeds of rice. Genetics.

[b19] Dai Y, Jia Y, Correll J, Wang X, Wang Y (2010). Diversification and evolution of the avirulence gene *AVR-Pita1* in field isolates of *Magnaporthe oryzae*. Fungal Genetics and Biology.

[b20] Dean R, Van Kan JA, Pretorius ZA, Hammond-Kosack KE, Di Pietro A, Spanu PD, Rudd JJ, Dickman M, Kahmann R, Ellis J (2012). The top 10 fungal pathogens in molecular plant pathology. Molecular Plant Pathology.

[b21] Don LD, Kusaba M, Urashima AS, Tosa Y, Nakayashiki H, Mayama S (1999a). Population structure of the rice blast fungus in Japan examined by DNA fingerprinting. Annals of the Phytopathological Society of Japan.

[b22] Don LD, Tosa Y, Nakayashiki H, Mayama S (1999b). Population structure of the rice blast fungus in Vietnam. Annals of the Phytopathological Society of Japan.

[b23] Dutech C, Fabreguettes O, Capdevielle X, Robin C (2010). Multiple introductions of divergent genetic lineages in an invasive fungal pathogen, *Cryphonectria parasitica*, in France. Heredity.

[b24] Estoup A, Guillemaud T (2010). Reconstructing routes of invasion using genetic data: why, how and so what?. Molecular Ecology.

[b25] Evanno G, Regnaut S, Goudet J (2005). Detecting the number of clusters of individuals using the software structure: a simulation study. Molecular Ecology.

[b26] Falush D, Stephens M, Pritchard J (2003). Inference of population structure using multilocus genotype data: linked loci and correlated allele frequencies. Genetics.

[b27] Fisher MC, Henk DA, Briggs CJ, Brownstein JS, Madoff LC, McCraw SL, Gurr SJ (2012). Emerging fungal threats to animal, plant and ecosystem health. Nature.

[b28] Fuentes JL, Correa-Victoria FJ, Escobar F, Mora L, Duque MC, Deus JE, Cornide MT (2003). Genetic diversity analysis of the rice blast pathogen population at two locations in Cuba. Biotecnología Aplicada.

[b29] Fuller DQ, Qin L, Zheng Y, Zhao Z, Chen X, Hosoya LA, Sun GP (2009). The domestication process and domestication rate in rice: spikelet bases from the Lower Yangtze. Science.

[b30] Garris AJ, Tai TH, Coburn J, Kresovich S, McCouch S (2005). Genetic structure and diversity in *Oryza sativa* L. Genetics.

[b31] Giraud T, Enjalbert J, Fournier E, Delmote F, Dutech C (2008). Population genetics of fungal diseases of plants. Parasite.

[b32] Gladieux P, Zhang XG, Afoufa-Bastien D, Valdebenito Sanhueza RM, Sbaghi M, Le Cam B (2008). On the origin and spread of the scab disease of apple: out of Central Asia. PLoS One.

[b33] Gomez-Alpizar L, Carbone I, Ristaino JB (2007). An Andean origin of *Phytophthora infestans* inferred from mitochondrial and nuclear gene genealogies. Proceedings of the National Academy of Sciences, USA.

[b34] González-Varela G, González AJ, Milgroom MG (2011). Clonal population structure and introductions of the chestnut blight fungus, *Cryphonectria parasitica*, in Asturias, northern Spain. European Journal of Plant Pathology.

[b35] Guillemaud T, Ciosi M, Lombaert E, Estoup A (2011). Biological invasions in agricultural settings: insights from evolutionary biology and population genetics. Comptes Rendus Biologies.

[b36] Hardy OJ, Vekemans X (2002). SPAGeDi: a versatile computer program to analyse spatial genetic structure at the individual or population levels. Molecular Ecology Notes.

[b37] Huang X, Kurata N, Wei X, Wang ZX, Wang A, Zhao Y, Liu K, Lu H, Li W, Guo Y (2012). A map of rice genome variation reveals the origin of cultivated rice. Nature.

[b38] Hufbauer RA, Facon B, Ravigné V, Turgeon J, Foucaud J, Lee CE, Rey O, Estoup A (2012). Anthropogenically induced adaptation to invade (AIAI): contemporary adaptation to human-altered habitats within the native range can promote invasions. Evolutionary Applications.

[b39] Hurles ME, Sykes BC, Jobling MA, Forster P (2005). The dual origin of the Malagasy in Island Southeast Asia and East Africa: evidence from maternal and paternal lineages. American Journal of Human Genetics.

[b40] Izawa T, Shimamoto K (1996). Becoming a model plant: the importance of rice to plant science. Trends in Plant Science.

[b41] Javan-Nikkah M, McDonald BA, Banke S, Hedjaroude GA (2004). Genetic structure of Iranian *Pyricularia grisea* populations based on re-PCR fingerprinting. European Journal of Phytopathology.

[b42] Jombart T, Devillard S, Balloux F (2010). Discriminant analysis of principal components: a new method for the analysis of genetically structured populations. BMC Genetics.

[b43] Kanzaki H, Yoshida K, Saitoh H, Fujisaki K, Hirabuchi A, Allaux L, Fournier E, Tharreau D, Terauchi R (2012). Arms race co-evolution of *Magnaporthe oryzae* AVR-Pik and rice Pik genes driven by their physical interactions. Plant Journal.

[b44] Kaye C, Milazzo J, Rozenfeld S, Lebrun MH, Tharreau D (2003). The development of simple sequence repeat markers for *Magnaporthe grisea* and their integration into an established genetic linkage map. Fungal Genetics and Biology.

[b45] Kumar J, Nelson RJ, Zeigler RS (1999). Population structure and dynamics of *Magnaporthe grisea* in the Indian Himalayas. Genetics.

[b46] Lawson Handley LJ, Estoup A, Evans DM, Thomas CE, Lombaert E, Facon B, Aebi A, Roy HE (2011). Ecological genetics of invasive alien species. BioControl.

[b47] Leberg PL (2002). Estimating allelic richness: effects of sample size and bottlenecks. Molecular Ecology.

[b48] Lee CE, Gelembiuk GW (2008). Evolutionary origins of invasive populations. Evolutionary Applications.

[b49] Leslie JF, Klein KK (1996). Female fertility and mating type effects on effective population size and evolution in filamentous fungi. Genetics.

[b50] Levy M, Correa FJ, Zeigler RS, Xu S, Hamer JE (1993). Genetic diversity of the rice blast fungus in a disease nursery in Colombia. Phytopathology.

[b51] Levy M, Romao J, Marchetti MA, Hamer JE (1991). DNA fingerprinting with a dispersed repeated sequence resolves pathotype diversity in the rice blast fungus. Plant Cell.

[b52] Lombaert E, Guillemaud T, Cornuet J-M, Malausa T, Facon B, Estoup A (2010). Bridgehead effect in the worldwide invasion of the biocontrol Harlequin ladybird. PLoS One.

[b53] Londo JP, Chiang YC, Hung KH, Chiang TY, Schaal BA (2006). Phylogeography of Asian wild rice, *Oryza rufipogon*, reveals multiple independent domestications of cultivated rice, *Oryza sativa*. Proceedings of the National Academy of Sciences, USA.

[b54] Montarry J, Andrivon D, Glais I, Corbiere R, Mialdea G, Delmotte F (2010). Microsatellite markers reveal two admixed genetic groups and an ongoing displacement within the French population of the invasive plant pathogen *Phytophthora infestans*. Molecular Ecology.

[b55] Nei M (1987). Molecular evolutionary genetics.

[b56] Nottéghem JL, Silué D (1992). Distribution of the mating type alleles in *Magnaporthe grisea* populations pathogenic on rice. Phytopathology.

[b57] Park SY, Milgroom MG, Han SS, Kang S, Lee YH (2003). Diversity of pathotypes and DNA fingerprint haplotypes in populations of *Magnaporthe grisea* in Korea over two decades. Phytopathology.

[b58] Park SY, Milgroom MG, Han SS, Kang S, Lee YH (2008). Genetic differentiation of *Magnaporthe oryzae* populations from scouting plots and commercial rice fields in Korea. Phytopathology.

[b59] Parks JC, Werth CR (1993). A study of spatial features of clones in a population of bracken fern, *Pteridium aquilinum**Dennstaedtiaceae*. American Journal of Botany.

[b60] Pennisi E (2010). Armed and dangerous. Science.

[b61] Piotti E, Rigano MM, Rodino D, Rodolfi M, Castiglione S, Picco AM, Sala F (2005). Genetic structure of *Pyricularia grisea* (Cooke) Sacc. Isolates from Italian paddy fields. Journal of Phytopathology.

[b62] Pritchard JK, Stephens P, Donnelly P (2000). Inference of population structure using multilocus genotype data. Genetics.

[b63] Raboin LM, Selvi A, Oliveira KM, Paulet F, Calatayud C, Zapater MF, Brottier P, Luzaran R, Garsmeur O, Carlier J (2007). Evidence for the dispersal of a unique lineage from Asia to America and Africa in the sugarcane fungal pathogen *Ustilago scitaminea*. Fungal Genetics and Biology.

[b64] Raymond M, Rousset F (1995). Genepop (version-1.2) – Population-genetics software for exact tests and ecumenicism. Journal of Heredity.

[b65] Robert S, Ravigne V, Zapater MF, Abadie C, Carlier J (2012). Contrasting introduction scenarios among continents in the worldwide invasion of the banana fungal pathogen *Mycosphaerella fijiensis*. Molecular Ecology.

[b66] Roumen E, Levy M, Nottéghem JL (1997). Characterisation of the European pathogen population of *Magnaporthe grisea* by DNA fingerprinting and pathotype analysis. European Journal of Plant Pathology.

[b67] Saleh D, Xu P, Shen Y, Li CY, Adreit H, Milazzo J, Ravigné V, Bazin E, Nottéghem JL, Fournier E (2012). Sex at the origin: an Asian population of the rice blast fungus *Magnaporthe oryzae* reproduces sexually. Molecular Ecology.

[b68] Schurko AM, Logsdon JM (2008). Using a meiosis detection toolkit to investigate ancient asexual “scandals” and the evolution of sex. BioEssays.

[b69] Shull V, Hamer J, Zeigler RS, Leong SA, Teng P (1994). Genome structure and variability in *Pyricularia grisea*. Rice blast disease.

[b70] Silué D, Nottéghem JL (1990). Production of perithecia of *Magnaporthe oryzae* on rice plants. Mycological Research.

[b71] Soubabère O, Dioh W, Lebrun MH, Nottéghem JL, Tharreau D, Tharreau D, Lebrun MH, Talbot NJ, Nottéghem JL (2000). Comparative continental variation of the rice blast fungus using Sequence Characterized Amplified Region markers. Advances in rice blast research.

[b72] Stenberg P, Lundmark M, Saura A (2003). MLGSim: a program for detecting clones using a simulation approach. Molecular Ecology Notes.

[b73] Stukenbrock EH, Banke S, Javan-Nikkhah M, McDonald BA (2007). Origin and domestication of the fungal wheat pathogen *Mycosphaerella graminicola* via sympatric speciation. Molecular Biology and Evolution.

[b74] Stukenbrock EH, McDonald BA (2008). The origins of plant pathogens in agro-ecosystems. Annual Review of Phytopathology.

[b75] Takahashi M, Ashizawa T, Hirayae K, Moriwaki J, Sone T, Sonoda R, Noguchi MT, Nagashima S, Ishikawa K, Arai M (2010). One of two major paralogs of *AVR-Pita1* is functional in Japanese rice blast isolates. Phytopathology.

[b76] Takan JP, Chipili J, Muthumeenakshi S, Talbot NJ, Manyasa EO, Bandyopadhyay R, Sere Y, Nutsugah SK, Talhinhas P, Hossain M (2012). *Magnaporthe oryzae* populations adapted to finger millet and rice exhibit distinctive patterns of genetic diversity, sexuality and host interaction. Molecular Biotechnologies.

[b77] Tharreau D, Fudal I, Andriantsimialona D, Santoso  , Utami D, Fournier E, Lebrun MH, Nottéghem JL, Wang GL, Valent B (2009). World population structure and migration of the rice blast fungus, *Magnaporthe oryzae*. Advances in genetics, genomics and control of rice blast disease.

[b78] Tibayrenc M, Kjellberg F, Ayala FJ (1990). A clonal theory of parasitic protozoa: the population structures of Entomœba, Giardia, Leishmania, Nægleria, Plasmodium, Trichomonas, and Trypanosoma and their medical and taxonomical consequences. Proceedings of the Natural Academy of Sciences, USA.

[b79] Valent B (1990). Rice blast as a model system for plant pathology. Phytopathology.

[b80] Valent B, Crawford MS, Weaver CG, Chumley FG (1986). Genetic studies of fertility and pathogenicity in *Magnaporthe grisea**Pyricularia oryzae*. Iowa State Journal of Research.

[b81] Weir BS, Cockerham CC (1984). Estimating *F*-statistics for the analysis of population structure. Evolution.

[b82] Xia JQ, Correll JC, Lee FN, Rhoads DD, Marchetti MA (1993). DNA fingerprint to examine variation in the *Magnaporthe grisea**Pyricularia grisea*) population in two rice fields in Arkansas. Phytopathology.

[b83] Xia JQ, Correll J, Lee FN, Ross WJ (2000). Regional population diversity of *Pyricularia grisea* in Arkansas and the influence of host selection. Plant Disease.

[b84] Xu JR, Hamer JE (1995). Assessment of *Magnaporthe grisea* mating type by spore PCR. Fungal Genetics Newsletter.

[b85] Zeigler RS (1998). Recombination in *Magnaporthe grisea*. Annual Review of Phytopathology.

[b86] Zeigler RS, Cuoc LX, Scott RP, Bernardo MA, Chen DH, Valent B, Nelson RJ (1995). The relationship between lineage and virulence in *Pyricularia grisea* in the Philippines. Phytopathology.

