# Supplementary material for: South-East Asia is the center of origin, diversity and dispersion of the rice blast fungus, Magnaporthe oryzae
Source: New Phytol. 2013 Dec 10;201(4):1440–56. doi: 10.1111/nph.12627 (PMC4265293; doi:10.1111/nph.12627)
Supplement: Fig S1 — Genetic structure of worldwide individuals of Magnaporthe oryzae: comparison between the DAPC and Structure methods. Fig. S2 Number of shared and specific alleles calculated over 10 microsatellites for the four Magnaporthe oryzae clusters identified in Asia. [file nph0201-1440-SD1.docx]

**Saleh *et al*. South-East Asia is the center of origin, diversity and dispersion of the rice blast fungus, *Magnaporthe oryzae*.**

**Supporting Information Figs S1-S2**

**Supporting Information Fig. S1. Genetic structure of 1372 worldwide individuals of *Magnaporthe oryzae*: comparison between the Discriminant Analysis on Principal Components (DAPC; first line) and the Bayesian method implemented in the software Structure (second line) without geographic prior.**

Assignments of individuals to each of the three clusters are symbolized by colors (A, red; B, green; C, blue). Each vertical bar represents an individual and the probability of assignment to a cluster is given by the length of the line of the corresponding color. Admixed individuals are represented by lines with two or three colors. Clone correction of the dataset did not change the results.


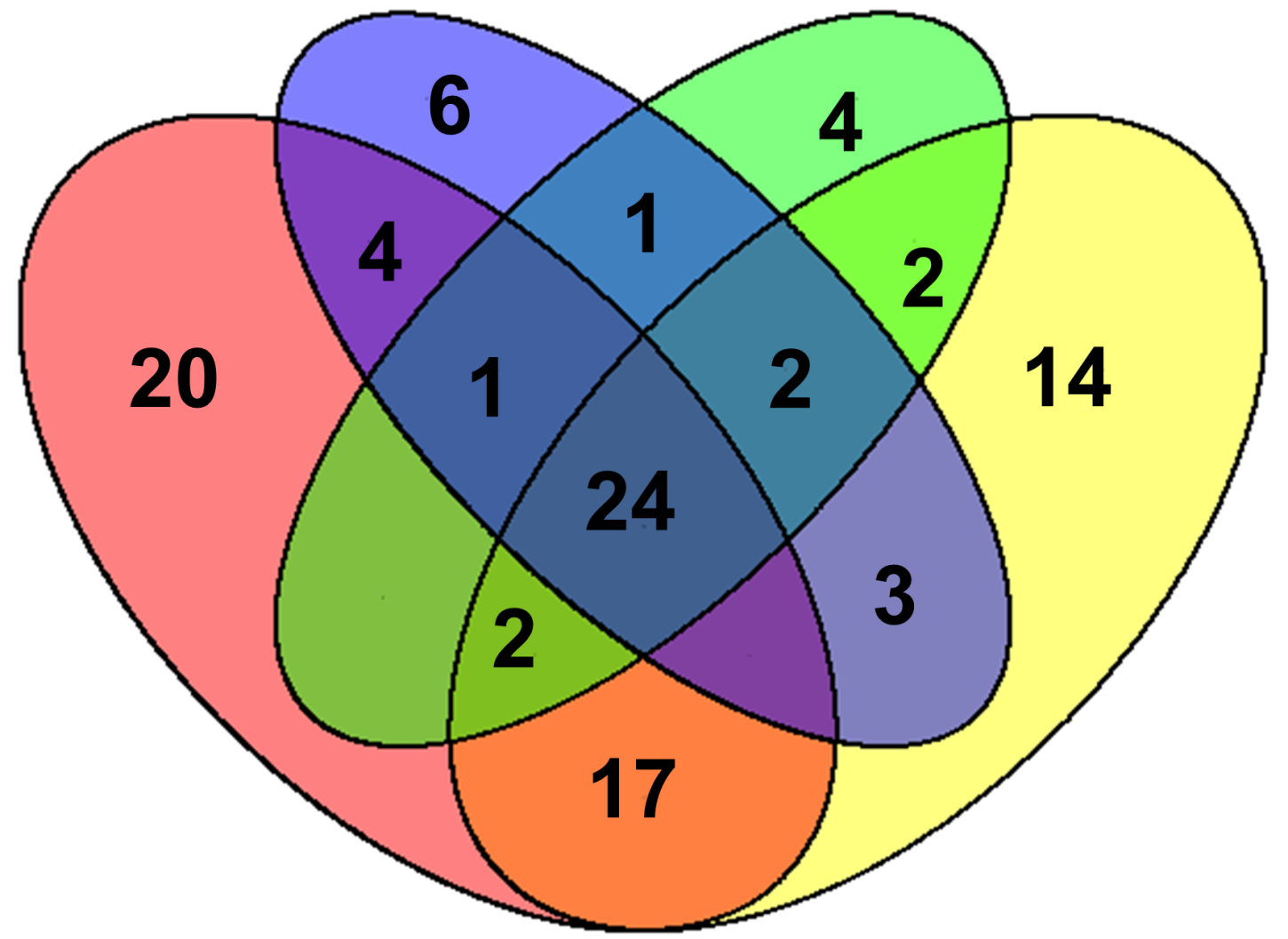


**Supporting Information Fig. S2. Venn diagram representing the number of shared and specific alleles calculated over 10 microsatellites loci for the four *Magnaporthe oryzae* clusters identified in Asia.** Yellow, cluster 1; green, cluster 2; blue, cluster 3; red, cluster 4.
