# Supplementary material for: South-East Asia is the center of origin, diversity and dispersion of the rice blast fungus, Magnaporthe oryzae
Source: New Phytol. 2013 Dec 10;201(4):1440–56. doi: 10.1111/nph.12627 (PMC4265293; doi:10.1111/nph.12627)
Supplement: Table S1 — Characteristics of the 10 microsatellites used for Magnaporthe oryzae genotyping Table S2 Unbiased gene diversity and number of private alleles in random subsamples performed in 46 Magnaporthe oryzae worldwide populations Table S3 Gene diversity and mean number of private alleles in subsamples of Magnaporthe oryzae individuals from the same genetic cluster in Asia Table S4 Information on Magnaporthe oryzae multilocus genotypes repeated within populations and shared between populations Table S5 Distribution of Magnaporthe oryzae individuals in clusters according to the type of rice culture [file nph0201-1440-SD2.doc]

**Saleh *et al*. South-East Asia is the center of origin, diversity and dispersion of the rice blast fungus, *Magnaporthe oryzae*.**

**Supporting Information Tables S1-S5**

| Marker name | Chromosome | Supercontig (Range on Supercontig pb) | Repeat motif | Primers sequences | *T_m_* (°C) | *N_t_* |
| --- | --- | --- | --- | --- | --- | --- |
| pyrms63-64 | 1 | 9 (243439-243601) | CT_15_ | F: (NED)-TTGGGATCTTCGGTAAGACG | 57 | 11 |
|  |  |  |  | R: GCCGACAAGACACTGAATGA |  |  |
| pyrms83-84b | 2 | 18 (211631-211742) | TCA_13_ | F: (PET)-GTCTGCCTCGACTCCTTCAC | 57 | 10 |
|  |  |  |  | R: GCAAAGTTGTTTGAGCAAGG |  |  |
| pyrms319-320 | 2 | 18 (52505-52795) | CAA_6_ | F: (NED)-TAAGACCACTGGCGGAATCT | 57 | 4 |
|  |  |  |  | R: GGCTTTGTCTGGTTGTACGG |  |  |
| pyrms77b-78 | 3 | 24 (510594-510813) | CA_24_ | F: (PET)-AGGCTCTCTGCCTACGAAGT | 57 | 14 |
|  |  |  |  | R: GCTTTCGGCAAGCCTAATC |  |  |
| pyrms607-608 | 3 | 28 (1095575-1095864) | GCA_13_ | F: (VIC)-CCCAAGCTCCATAATACGCTAC | 57 | 9 |
|  |  |  |  | R: TCCGAGACTCTTTGGATAGCAC |  |  |
| pyrms37-38 | 4 | 15 (1042756-1042961) | CA_6_+CT_12_ | F: (NED)-ACCCTACCCCCACTCATTTC | 57 | 6 |
|  |  |  |  | R: AGGATCAGCCAATGCCAAGT |  |  |
| pyrms47-48 | 4 | 12 (707029-707203) | TA_15_ | F: (FAM)-TCACATTTGCTTGCTGGAGT | 57 | 12 |
|  |  |  |  | R: AGACAGGGTTGACGGCTGAA |  |  |
| pyrms233-234 | 5 | 10 (38361-38617) | CAG_10_ | F: (FAM)-TGAGATGGACCGCATGATTA | 57 | 14 |
|  |  |  |  | R: TTGATGGCAGAGACATGAGC |  |  |
| pyrms427-428 | 5 | 13 (331827-332045) | AT_16_ | F: (VIC)-CTGTCACCACAACCAAGACG | 57 | 17 |
|  |  |  |  | R: TTGCCCTGATTTGTCAGTCA |  |  |
| pyrms657-658 | 6 | 21 (3745780-3745947) | CA_12_ | F: (VIC)-ATCAGTCGAACCCACAAAGC | 57 | 4 |
|  |  |  |  | R: ATGTGTGGACGAACCAGTCC |  |  |
| pyrms385-386 | 7 | 23 (2709066-2709228) | TAG_9_ | F: (VIC)-CCTTGTTTTCCCCCTGTGTA | 57 | 9 |
|  |  |  |  | R: TGGGAAGAAGAGACCGAAGA |  |  |

**Supporting Information Table S1: Characteristics of the 10 microsatellite markers used for *Magnaporthe oryzae* genotyping. Marker name, position, repeat motif, primer sequence, melting temperature (*T_m_*) and total number of alleles (*N_t_*) for 17 microsatellite markers tested on all strains genotyped.**

| *Area* | Country | population | N | *H_n.b._* | | *N_p_* | |
| --- | --- | --- | --- | --- | --- | --- | --- |
|  |  |  |  | observed | sampled | observed | sampled |
| *Asia* | China | CH1 | 107 | 0.6 | 0.6 ± 0.02 | 1.60 | 0.42 ± 0.16 |
|  |  | CH2 | 38 | 0.5 | 0.5 ± 0.03 | 0.20 | 0.02 ± 0.04 |
|  |  | CH3 | 23 | 0.5 | 0.5 ± 0.03 | 0.10 | 0.02 ± 0.04 |
|  |  | CH4 | 25 | 0.1 | 0 ± 0.02 | 0 | 0 ± 0 |
|  |  | CH5 | 28 | 0.5 | 0.5 ± 0.03 | 0 | 0 ± 0 |
|  |  | CH6 | 30 | 0.3 | 0.3 ± 0.08 | 0 | 0 ± 0 |
|  |  | CH7 | 14 | 0.3 | 0.2 ± 0.03 | 0 | 0 ± 0 |
|  | Indonesia | ID1 | 20 | 0.3 | 0.3 ± 0.02 | 0.10 | 0.02 ± 0.04 |
|  |  | ID2 | 19 | 0.1 | 0.1 ± 0.03 | 0.10 | 0 ± 0 |
|  |  | ID3 | 16 | 0.1 | 0.1 ± 0.03 | 0 | 0 ± 0 |
|  | Laos | LA1 | 15 | 0.6 | 0.6 ± 0.01 | 0.40 | 0.24 ± 0.13 |
|  |  | LA2 | 9 | 0.5 | - | 0.20 | - |
|  | Nepal | NP1 | 31 | 0.2 | 0.2 ± 0.05 | 0.10 | 0.02 ± 0.04 |
|  |  | NP2 | 15 | 0.4 | 0.4 ± 0.1 | 0.30 | 0.18 ± 0.11 |
|  |  | NP3 | 6 | 0.5 | - | 0.10 | - |
|  | Thailand | TH | 27 | 0.5 | 0.4 ± 0.03 | 0.10 | 0.08 ± 0.04 |
| *Europe / Mediterranean basin* | France | FR1 | 23 | 0.1 | 0.1 ± 0.02 | 0 | 0 ± 0 |
|  |  | FR2 | 17 | 0.2 | 0.1 ± 0.02 | 0 | 0 ± 0 |
|  |  | FR3 | 18 | 0.1 | 0.1 ± 0.02 | 0 | 0 ± 0 |
|  |  | FR4 | 17 | 0.1 | 0.1 ± 0.04 | 0 | 0 ± 0 |
|  |  | FR5 | 22 | 0 | 0 ± 0.01 | 0.10 | 0.06 ± 0.05 |
|  |  | FR6 | 37 | 0.3 | 0.3 ± 0.06 | 0.10 | 0 ± 0 |
|  |  | FR7 | 15 | 0 | 0 ± 0.01 | 0 | 0 ± 0 |
|  | Greece | GR1 | 10 | 0.2 | - | 0 | - |
|  |  | GR2 | 10 | 0.2 | - | 0 | - |
|  |  | GR3 | 10 | 0.2 | - | 0 | - |
|  |  | GR4 | 9 | 0.1 | - | 0 | - |
|  |  | GR5 | 9 | 0.2 | - | 0 | - |
|  |  | GR6 | 10 | 0.2 | - | 0 | - |
|  |  | GR7 | 9 | 0.1 | - | 0 | - |
|  | Hungary | HN1 | 7 | 0.1 | - | 0 | - |
|  |  | HN2 | 3 | 0.1 | - | 0 | - |
|  |  | HN3 | 7 | 0.1 | - | 0 | - |
|  | Morocco | MC | 15 | 0.2 | 0.2 ± 0.04 | 0.10 | 0.1 ± 0 |
|  | Spain | SP1 | 12 | 0.3 | 0.3 ± 0.02 | 0 | 0 ± 0 |
|  |  | SP2 | 31 | 0.2 | 0.2 ± 0.05 | 0 | 0 ± 0 |
|  |  | SP3 | 11 | 0.3 | 0.3 ± 0.01 | 0 | 0 ± 0 |
|  |  | SP4 | 22 | 0.3 | 0.2 ± 0.04 | 0 | 0 ± 0 |
|  |  | SP5 | 13 | 0 | 0 ± 0.01 | 0 | 0 ± 0 |
|  |  | SP6 | 9 | 0.1 | - | 0 | - |
|  |  | SP7 | 10 | 0 | - | 0 | - |
|  |  | SP8 | 18 | 0.1 | 0.1 ± 0 | 0 | 0 ± 0 |
|  |  | SP9 | 29 | 0.1 | 0.1 ± 0.01 | 0.10 | 0.04 ± 0.05 |
|  | Turkey | TR | 19 | 0 | 0 ± 0.02 | 0 | 0 ± 0 |
| *Americas* | Colombia | CL1 | 17 | 0.1 | 0.1 ± 0.05 | 0 | 0 ± 0 |
|  |  | CL2 | 31 | 0.1 | 0 ± 0.02 | 0.10 | 0.04 ± 0.05 |
|  | French Guyana | GY | 12 | 0.2 | 0.2 ± 0.07 | 0.20 | 0.14 ± 0.09 |
|  | USA | USA1 | 37 | 0.6 | 0.5 ± 0.04 | 0.10 | 0.08 ± 0.04 |
|  |  | USA2 | 39 | 0 | 0 ± 0.02 | 0 | 0 ± 0 |
| *Madagascar* | Madagascar | MD1 | 264 | 0 | 0 ± 0.04 | 0.30 | 0 ± 0 |
|  |  | MD2 | 27 | 0.2 | 0.2 ± 0.03 | 0 | 0 ± 0 |
|  |  | MD3 | 37 | 0.1 | 0.1 ± 0.02 | 0 | 0 ± 0 |
|  |  | MD4 | 15 | 0 | 0 ± 0 | 0 | 0 ± 0 |
|  |  | MD5 | 23 | 0.2 | 0.2 ± 0.03 | 0 | 0 ± 0 |
|  |  | MD6 | 25 | 0.1 | 0.1 ± 0.03 | 0 | 0 ± 0 |

**Supporting Information Table S2.** **Unbiased gene diversity (*H_n.b._*), and number of private alleles (*N_p_*), in five random subsamples of 10 individuals performed in each of the 46 *Magnaporthe oryzae* worldwide populations for which sample size was higher than 10 (see Table1).** Sampling was not performed in populations of size inferior to 10 (shaded in grey).

|  | Cluster 1 | |  | Cluster 2 | |  | Cluster 3 | |  | Cluster 4 | |
| --- | --- | --- | --- | --- | --- | --- | --- | --- | --- | --- | --- |
| Population | *H_n.b._* | *N_p_* |  | *H_n.b._* | *N_p_* |  | *H_n.b._* | *N_p_* |  | *H_n.b._* | *N_p_* |
| CH1 | 0.53 | 0.3 |  | - | - |  | - | - |  | 0.57 | 1.3 |
| LA1 | 0.36 | 0.3 |  | - | - |  | - | - |  | 0.44 | 0.1 |
| CH5 | 0.50 | - |  | 0.42 | - |  | 0.23 | - |  | - | - |
| CH2 | 0.06 | - |  | 0.36 | 0.1 |  | 0.23 | - |  | - | 0.1 |
| LA2 | - | 0.2 |  | - | - |  | - | - |  | 0.51 | - |
| CH3 | 0.19 | - |  | - | - |  | 0.20 | - |  | - | 0.1 |
| NP3 | 0.49 | 0.1 |  | - | - |  | - | - |  | - | - |
| TH | 0.21 | 0.1 |  | - | - |  | - | - |  | 0.40 | - |
| NP2 | - | 0.3 |  | - | - |  | 0.07 | - |  | - | - |
| ID1 | - | - |  | - | - |  | 0.28 | 0.1 |  | - | - |
| CH7 | - | - |  | - | - |  | 0.21 | - |  | - | - |
| CH6 | - | - |  | 0.10 | - |  | - | - |  | - | - |
| NP1 | 0.13 | 0.1 |  | - | - |  | - | - |  | - | - |
| ID3 | - | - |  | - | - |  | 0.11 | - |  | - | - |
| ID2 | - | - |  | - | - |  | 0.09 | 0.1 |  | - | - |
| CH4 | - | - |  | 0.05 | - |  | - | - |  | - | - |

**Supporting Information Table S3**. **Gene diversity (*H_n.b._*) and mean number of private alleles (*N_p_*) in subsamples of *Magnaporthe oryzae* individuals from the same genetic cluster in Asia.**

| **MLG** | **Populations** | **Number of repeats within populations** | ***P_sex_*** | **P-value** |
| --- | --- | --- | --- | --- |
| M1 | CH2 | 8 | 6.39 e-14 | 0 |
|  | CH7 | 4 | 4.05 e-04 | 0 |
| M2 | CH2 | 2 | 2.13 e-04 | 0 |
|  | CH7 | 1 | - | - |
|  | NP1 | 1 | - | - |
| M3 | CH3 | 1 | - | - |
|  | CH5 | 2 | 4.31 e-12 | 0 |
| M4 | CH3 | 1 | - | - |
|  | CH4 | 4 | 0.73 | 0 |
|  | CH6 | 18 | 1.22 e-07 | 0 |
|  | HN1 | 8 | 0.45 | 0 |
| M5 | CH5 | 1 | - | - |
|  | CH6 | 3 | 9.46 e-10 | 0 |
| M6 | CH2 | 1 | - | - |
|  | CH3 | 6 | 6.99 e-07 | 0 |
|  | CH7 | 1 | - | - |
| M7 | CH2 | 1 | - | - |
|  | CH7 | 1 | - | - |
| M8 | ID1 | 3 | 0.02 | 0 |
|  | ID2 | 7 | 0.44 | 0 |
|  | CH2 | 2 | 9.24 e-04 | 0 |
|  | CH5 | 5 | 1.66 e-10 | 0 |
|  | CH6 | 3 | 3.44 e-13 | 0 |
|  | CH7 | 3 | 0.17 | 0 |
|  | CL1 | 3 | 0.74 | 0 |
| M9 | ID2 | 1 | - | - |
|  | MD2 | 1 | - | - |
| M10 | ID1 | 1 | - | - |
|  | ID2 | 2 | 0.71 | 0 |
|  | ID3 | 1 | - | - |
|  | CH7 | 1 | - | - |
|  | CL1 | 1 | - | - |
| M11 | FR1 | 12 | 0.17 | 0 |
|  | FR3 | 1 | - | - |
|  | FR6 | 1 | - | - |
|  | FR7 | 11 | 0.68 | 0 |
|  | GR2 | 3 | 0.03 | 0 |
| M12 | FR1 | 3 | 0.92 | 0 |
|  | FR7 | 2 | 0.62 | 0 |
| M13 | FR1 | 1 | - | - |
|  | SP2 | 1 | - | - |
|  | SP5 | 10 | 0.68 | 0 |
|  | SP6 | 5 | 0.63 | 0 |
|  | SP7 | 3 | 0.62 | 0 |
|  | SP8 | 7 | 0.60 | 0 |
| M14 | FR2 | 5 | 0.77 | 0 |
|  | FR3 | 8 | 0.54 | 0 |
|  | FR4 | 9 | 0.45 | 0 |
|  | FR6 | 12 | 2.52 e-11 | 0 |
| M15 | FR2 | 4 | 0.10 | 0 |
|  | FR3 | 6 | 0.37 | 0 |
|  | FR4 | 2 | 0.74 | 0 |

**Supporting Information Table S4**. **Information on *Magnaporthe oryzae* multilocus genotypes (MLG) repeated within populations and shared between populations.** MLG shared between countries are shaded.

| **MLG** | **Populations** | **Number of repeats within populations** | ***P_sex_*** | **P-value** |
| --- | --- | --- | --- | --- |
| M16 | FR2 | 1 | - | - |
|  | FR3 | 1 | - | - |
|  | FR4 | 1 | - | - |
|  | GR2 | 1 | - | - |
| M17 | FR2 | 2 | 0.37 | 0 |
|  | FR3 | 1 | - | - |
|  | TR | 1 | - | - |
| M18 | FR4 | 1 | - | - |
|  | GR5 | 1 | - | - |
| M19 | FR5 | 15 | 0.7 | 0 |
|  | FR6 | 2 | 0.48 | 0 |
|  | GR6 | 1 | - | - |
| M20 | FR6 | 11 | 4.51 e-09 | 0 |
|  | GR1 | 2 | 5.61 e-03 | 0 |
|  | GR2 | 1 | - | - |
|  | GR3 | 4 | 6.32 e-03 | 0 |
|  | GR4 | 1 | - | - |
|  | GR7 | 4 | 0.082 | 0 |
|  | SP1 | 1 | - | - |
|  | SP5 | 1 | - | - |
| M21 | FR6 | 2 | 4.27 e-06 | 0 |
|  | SP2 | 4 | 1.86 e-08 | 0 |
|  | SP3 | 3 | 2.12 e-04 | 0 |
|  | SP4 | 2 | 2.02 e-03 | 0 |
|  | SP9 | 14 | 0.64 | 0 |
| M22 | GR1 | 1 | - | - |
|  | GR4 | 1 | - | - |
|  | GR6 | 2 | 0.24 | 0 |
| M23 | GR1 | 3 | 0.03 | 0 |
|  | GR2 | 3 | 0.11 | 0 |
|  | GR3 | 3 | 0.06 | 0 |
|  | GR7 | 4 | 0.16 | 0 |
| M24 | GR2 | 1 | - | - |
|  | GR3 | 1 | - | - |
|  | SP1 | 4 | 8.79 e-03 | 0 |
| M25 | GR1 | 1 | - | - |
|  | GR4 | 7 | 0.14 | 0 |
|  | GR5 | 6 | 0.04 | 0 |
|  | GR6 | 6 | 0.03 | 0 |
| M26 | HN1 | 1 | - | - |
|  | HN2 | 2 | 0.74 | 0 |
|  | HN3 | 3 | 0.60 | 0 |
|  | CH4 | 1 | - | - |
| M27 | SP1 | 2 | 0.22 | 0 |
|  | SP2 | 4 | 0.06 | 0 |
|  | SP3 | 1 | - | - |

**Supporting Information Table S4**. Continued.

| **MLG** | **Populations** | **Number of repeats within populations** | ***P_sex_*** | **P-value** |
| --- | --- | --- | --- | --- |
| M28 | SP2 | 3 | 0.05 | 0 |
|  | SP3 | 3 | 7.37 e-03 | 0 |
|  | SP4 | 5 | 1.69 e-03 | 0 |
|  | CH4 | 1 | - | - |
| M29 | SP2 | 10 | 0.05 | 0 |
|  | SP3 | 3 | 0.07 | 0 |
|  | SP4 | 5 | 0.01 | 0 |
|  | SP5 | 1 | - | - |
|  | SP6 | 4 | 0.63 | 0 |
|  | SP7 | 7 | 0.65 | 0 |
|  | SP8 | 7 | 0.60 | 0 |
| M30 | SP2 | 1 | - | - |
|  | USA2 | 25 | 0.44 | 0 |
| M31 | GY | 8 | 3.13 e-03 | 0 |
|  | TH | 1 | - | - |
| M32 | MD1 | 202 | 5.24 e-03 | 0 |
|  | MD3 | 17 | 0.34 | 0 |
|  | MD4 | 13 | 1 | 0 |
|  | MD5 | 1 | - | - |
|  | MD6 | 6 | 0.65 | 0 |
| M33 | MD1 | 1 | - | - |
|  | MD6 | 1 | - | - |
| M34 | MD1 | 1 | - | - |
|  | MD3 | 1 | - | - |
| M35 | MD1 | 4 | 0.75 | 0 |
|  | MD2 | 2 | 0.18 | 0 |
|  | MD3 | 5 | 0.28 | 0 |
| M36 | MD1 | 1 | - | - |
|  | MD3 | 1 | - | - |
| M37 | MD1 | 1 | - | - |
|  | MD3 | 1 | - | - |
| M38 | MD2 | 7 | 0.25 | 0 |
|  | MD5 | 3 | 0.75 | 0 |
|  | MD6 | 3 | 0.42 | 0 |
| M39 | MD2 | 2 | 0.14 | 0 |
|  | MD3 | 1 | - | - |
| M40 | MD1 | 1 | - | - |
|  | MD2 | 4 | 0.12 | 0 |
|  | MD5 | 7 | 0.27 | 0 |
|  | MD6 | 1 | - | - |
| M41 | MD1 | 1 | - | - |
|  | MD3 | 1 | - | - |

**Supporting Information Table S4.** Continued.

**a**

**b**

**Supporting Information Table S5.** **Distribution of *Magnaporthe oryzae* individuals in clusters (inferred using DAPC) according to the type of rice culture. a.** At the Asian scale. **b.** At the global scale. Expected values were calculated for an independent assortment on the overall frequencies of the different characters.
